# Metal Cation Detection in Drinking Water

**DOI:** 10.3390/s19235134

**Published:** 2019-11-23

**Authors:** Johnson Dalmieda, Peter Kruse

**Affiliations:** Department of Chemistry and Chemical Biology, McMaster University, Hamilton, ON L8S 4M1, Canada; dalmiej@mcmaster.ca

**Keywords:** water quality, chemical sensors, cations, heavy metals, lead, mercury

## Abstract

Maintaining a clean water supply is of utmost importance for human civilization. Human activities are putting an increasing strain on Earth’s freshwater reserves and on the quality of available water on Earth. To ensure cleanliness and potability of water, sensors are required to monitor various water quality parameters in surface, ground, drinking, process, and waste water. One set of parameters with high importance is the presence of cations. Some cations can play a beneficial role in human biology, and others have detrimental effects. In this review, various lab-based and field-based methods of cation detection are discussed, and the uses of these methods for the monitoring of water are investigated for their selectivity and sensitivity. The cations chosen were barium, cadmium, chromium, copper, hardness (calcium, magnesium), lead, mercury, nickel, silver, uranium, and zinc. The methods investigated range from optical (absorbance/fluorescence) to electrical (potentiometry, voltammetry, chemiresistivity), mechanical (quartz crystal microbalance), and spectrometric (mass spectrometry). Emphasis is placed on recent developments in mobile sensing technologies, including for integration into microfluidics.

## 1. Introduction

Water is an important part of human physiology. It is not only necessary for various bodily functions; it has various uses from agriculture to cleaning, food processing, chemical processes, and many more. The human body consists of 50% to 70% of water, and water intake is necessary for adequate kidney health. To maintain proper hydration, humans need at least one liter of water a day [1]. Water is also necessary for irrigation, and up to 42% of water in the United States is used for that purpose [2]. Together with soap, water is used to maintain personal hygiene and general cleanliness. Water is used for various cooking methods such as boiling or simmering (in its liquid state) or steaming (in its gaseous state as steam) [3]. In the chemical industry, water is used as a common solvent for aqueous soluble reagents due to its ability to dissolve many ionic species. Water vapor can also be used in some processes, such as the production of acrylic acid [4]. Since water is used in so many different ways around the world, it is important to maintain water to a safe standard for consumption and for use in the fields where it is required. Many countries and international agencies have guidelines for various parameters to maintain. In this review, the importance of cation maintenance and detection is discussed. Maximum allowable concentration (MAC) guideline values are listed in Table 1 for various jurisdictions.

Cations in water serve an important physiological purpose in humans. Elements such as copper, iron, calcium, magnesium, and many more contribute to various biological processes such as oxygen transport, enzymatic catalysis, DNA synthesis, and cell maturation. Many of these elements are provided through food intake; however, for areas in which meat consumption is low, the most abundant source of these elements is water [5]. Conversely, many other elements such as silver, lead, mercury, and cadmium can have detrimental effects on humans, such as nerve damage, growth defects, and even death.

There are many different methods used to determine the concentration of cations present in water. These methods can be divided into four categories: mechanical, optical, electrochemical, and spectroscopic/spectrometric. Mechanical methods use the mass or physical change an analyte induces to generate a signal that can correlate to the concentration. This can be done by using a piezoelectric material or through swelling of a film [6]. This allows for a very simple set-up in terms of portability and signal output but may not be very selective to the target analyte. Optical methods rely on the visual change in the properties of the sensor, whether it is a change in color, induced fluorescence of a non-fluorescent material, or quenching of fluorescence of a naturally fluorescing material. Almost all chemosensors follow the same formula in their molecular design: a receptor specific to the analyte, and a portion that generates a colorimetric signal [7]. This method is quick and selective and can be performed in situ. However, it lacks the sensitivity of more elaborate techniques. Electrochemical sensors induce a change in electrical property (current or voltage) proportional to the amount of analyte in solution. This allows for sensitive and selective quantification for many analytes in water, while still being quite portable [8]. Spectroscopic/spectrometric methods utilize the characteristic response of each atom to electromagnetic radiation to determine concentrations of those analytes in solution. This method is highly sensitive and selective but lacks the portability as it requires elaborate instrumentation [9].

As a range of methods are becoming available for water quality monitoring, we review state-of-the-art research that was recently published in the area of cation monitoring for aqueous environments. For the purpose of this review, we firstly discuss each detection method in some detail, followed by individual sections for the most important cationic species of relevance to water quality monitoring.

## 2. Methods

### 2.1. Mechanical

#### Quartz Crystal Microbalance (QCM)

QCM measures changes in mass by measuring changes in the frequency of a quartz crystal resonator. This change in frequency occurs due to the piezoelectric effect, which is the generation of electricity as a response to mechanical stress, or vice versa. The measurement occurs through a change in the resonance frequency caused by deposition of the analyte onto the surface of the quartz crystal. By functionalizing the surface of the quartz disc, selectivity to a particular analyte is achieved, and its binding affinity can be determined [10]. As the analyte adsorbs onto the piezoelectric crystal, the change in mass causes a change in the resonance frequency of the crystal. This relationship is defined by the Sauerbrey equation (Equation (1)), which indicates that the correlation between the change in mass and the change in frequency is linear [11].

(1) $$\Delta f=-\frac{2f_{0}^{2}}{A\sqrt{\rho_{q}\mu_{q}}}\Delta m,$$

where **Δ*f*** is the change in frequency, ***f***_0_ is the resonant frequency of the quartz crystal, **Δ*m*** is the change in mass, A is the active area of the crystal, ***ρ_q_*** is the density of quartz (2.65 g/cm^3^), and ***μ_q_*** is the shear modulus (2.95 × 10^11^ g/cm·s^2^). For a quartz crystal with a resonance frequency in the MHz range, changes in frequency can be measured with a sensitivity of 1 Hz [12]. The Sauerbrey equation assumes that depositing a certain mass of the analyte onto the crystal is equivalent to the crystal itself increasing in mass. This means that the equation is subject to three constraints: the adsorbed analyte remains rigid on the film once adsorbed, the mass of the analyte is small compared to the crystal itself, and the analyte is evenly distributed onto the crystal [13]. In liquid media, adsorption of the analyte may not fully couple to the frequency of the crystal, which leads to losses in energy. This loss in energy is due to the fact that, in liquids, the analyte forms softer films that do not remain rigid on the surface of the quartz crystal, which leads to non-resonant oscillations. In this case, the change in the rate of energy transfer (dissipation) from the crystal to the analyte also needs to be considered. This technique is known as QCM-D [14]. By taking the change in dissipation into account, information about the adsorbed analyte’s density, thickness, viscosity, and elasticity can also be known. To do this, the Voigt viscoelastic model is used, which relates the change in frequency and the change in dissipation to the viscoelastic properties of the adsorbed analyte and the liquid medium [15]. The ratio of the changes in dissipation compared to frequency can be used to determine the rigidity of the adsorbed analyte onto the quartz crystal. Ideally, a film would be considered viscoelastic if there is no change in dissipation. However, in real samples there are criteria, such as a Δ*D*/Δ***f*** ratio (the ratio of change in dissipation to the frequency change) of 10^−8^ Hz^−1^. Below this ratio, the film would be considered rigid, and, above this, it would be considered viscoelastic [16]. A typical QCM consists of a quartz disc with electrodes attached to the front and the back, an oscillator which oscillates at the resonant frequency, and a monitor that shows real-time frequency changes. As the analyte adsorbs onto the quartz crystal, the change in mass results in an electrical signal, which is output onto a monitor.

The advantage of QCM lies in its portability, its quick and sensitive response, and its ability to be functionalized to improve selectivity. However, non-specific adsorption or physisorption can lead to interference within the signal, decreasing its effectiveness in more complex matrices such as environmental samples.

### 2.2. Optical

#### 2.2.1. Colorimetry

Colorimetry is one of the most widely used analytical methods in chemistry. The main principle of colorimetry focuses on the detection of analyte based on the color change of the analyte solution or a surface (such as litmus paper). Detection is performed in various ways, such as with the eye or with instrumentation (such as a UV/vis spectrophotometer). Sensitivity of detection can range from a simple yes/no response to a proportional visual response or a change in concentration [17]. The concentration of an analyte can be determined by comparing the absorbance of a colored solution of analyte at a specific wavelength to a blank or a control solution. Solutions with known concentrations of analyte can be used to create a calibration equation which follows the Beer–Lambert law (Equation (2)).

(2) A=εlc,

where *A* is the absorbance of the sample, *l* is the path length of the cuvette, and c is the concentration of analyte in sample. The molar extinction coefficient, *ε*, can be obtained using the results from the calibration given that the concentration, *c*, and the path length (distance that the incident light travels through the sample, usually the diameter of the analyte containing cuvette), *l*, are known [18]. The concentration of an unknown analyte sample can now be determined using the obtained function.

Colorimetry is probably the easiest of all the methods for implementation, since it gives a visual indication of the presence of target analytes in a sample. This method is highly portable and inexpensive (some colorimetry kits can be bought at retail stores), and it may not require any additional instrumentation. However, for quantitative measurements, a UV/vis spectrophotometer is required, which substantially increases cost. In addition, the color change that occurs may only be detectable at higher concentrations (in some cases, much higher than regulation), meaning that this method lacks sensitivity. The color change may also be induced by interferences in the sample matrix, affecting the selectivity of the method.

#### 2.2.2. Fluorescence

In contrast to colorimetry, which measures the decrease in transmission of the incident light, fluorescence spectroscopy measures the increase in the emission of light as a result of excitation. Fluorescence occurs when an excited electron relaxes back to its ground state, emitting a photon [19]. The emitted photon can have an energy that is lower than the excitation energy (Stokes shift due to vibrational relaxation), higher than the excitation energy (two-photon absorption leading to a transition equal to the sum of the two photons), or the same as the excitation energy (resonance fluorescence) [20]. Fluorescence also has a certain efficiency associated with it, known as its quantum yield. Quantum yield is the ratio of the photons emitted by the analyte to the photons absorbed by the analyte. This can give important information about the number of molecules in the solution interacting with the incident photons [21]. Through the binding of the selected analyte to a fluorescent compound, one can detect he concentration of the analyte quite accurately based on the degree of quenching of the fluorescence signal (decrease in quantum yield). By using a fluorometer with a single exciting and detection wavelength, one can monitor changes in quantum efficiency and correlate them to concentrations of analyte in solution [22]. Compared to colorimetry, fluorescence can also be used in bioimaging and in intracellular detection [23].

Many of the advantages and drawbacks listed for colorimetry also apply to fluorescence. The main difference between the two, however, is that fluorescence requires a light source to excite the analyte within the sample. This light source may lead to other interferences in the sample becoming excited as well, affecting the quantum yield of the analyte in the sample. To avoid this, a sample workup step may be required.

#### 2.2.3. Surface-Enhanced Raman Spectroscopy (SERS)

SERS enhances the Raman scattering of molecules by having those molecules adsorbed onto metal or organic surfaces [24]. There is debate on the nature of the SERS effect; however, the strongest theory for its mechanism is the electromagnetic (EM) theory. This theory states that the enhancement in the signal is caused by surface plasmon resonance (SPR). When the incident light impacts the analyte adsorbed onto the surface, the collective electronic states at the surface (plasmons) become excited. Enhancement of the EM field (from the incident light) is at its highest when the frequency of the surface plasmon oscillations is equal to the frequency of the incident light [25]. This field enhancement increases the intensity of the incident light, which enhances the Raman signal. The oscillations that occur on the surface also enhance the Raman signal. The electric field is enhanced by a factor of two at each step; thus, the signal is enhanced by a power of four [26]. Samples for SERS are commonly prepared by depositing the analyte solution onto a dielectric with a noble-metal surface (usually silver or gold nanoparticles) [27]. An important property of the surface is the structure and size of the nanoparticles themselves. For ideal enhancement, the surface must be uniform. If the nanoparticles are too big, higher-order transitions may lead to non-radiative scattering, decreasing the enhancement. If they are too small, they may not have the ability to oscillate upon the impact of incident light. When combined with SPR, SERS can be used to identify and quantify analytes in liquids [28].

SERS allows for quantification of analytes at ultra-trace levels due to the multiplying effect of the analyte on the enhancement of the surface plasmon resonance. The method is also quite portable and can be used for in situ analysis. However, the plasmonic material used (usually gold or silver) is expensive and inaccessible for wide-scale use. 

#### 2.2.4. Atomic Absorption Spectroscopy (AAS)

The principle behind AAS is that the wavelength of the optical absorption of the analyte in the gas phase provides the identity of the analyte, while the intensity of the absorbance correlates to the concentration of that analyte in the sample. Unlike the colorimetric absorbance method, the sample is atomized first before being exposed to light. Various methods exist to atomize the sample, the most common being flame atomizers and electrothermal atomizers. When using flame atomizers, a nebulizer turns the liquid or gaseous samples into a fine mist. They are then subjected to mixture with a combustible gas, which produces a flame containing the atomic aerosol for analysis [29]. Electrothermal atomization utilizes a graphite furnace which contains a graphite tube that has a small cavity for holding the sample. Through the Joule effect, the graphite tube is heated (and can reach temperatures of 3000 K). This decomposes and atomizes the sample for analysis. Due to the high atom density, the graphite furnace has a higher sensitivity than the flame atomizer [30]. For some heavy metals, it is difficult to reduce them to their elemental state through flame or high heat. In these cases, the sample needs to be reacted with a reducing agent before analysis (a metal hydride is usually formed). These metallic hydrides then thermalize at 1000 K. This is used for samples such as arsenic and mercury. Measurements are made using the Beer–Lambert law (Equation (2)). A calibration curve can be prepared with the target analyte at the specific wavelength for the analyte. This provides the correction coefficient for the unknown sample (there is no molar extinction coefficient since there is no cuvette length; thus, these two parameters are replaced by a correction factor k). The concentration of the analyte in the unknown sample can then be determined using this correction factor and the obtained absorbance.

This method allows for detection at ultra-trace levels of analyte, and, since each atom has a specific wavelength on the spectrum, interference is eliminated, making this a highly selective and sensitive method which is also capable of simultaneous detection. However, AAS instrumentation is quite large and, therefore, cannot be used outdoors in the field. The method is also destructive to the samples, since analysis requires the sample to be atomized.

### 2.3. Electrical

#### Chemiresistivity/ChemFET (Chemical Field Effect Transistor)

Chemiresistivity is a change in resistance of a conductive material due to a change in its chemical environment. This change in resistance can be determined by placing contacts at either end of a thin film made from the material to measure the current passing through the material at a given voltage [31]. The basic components of a chemiresistor are a conducting material, the contacts at either end to facilitate conduction, and an electrical readout device which displays changes in the resistance of the conducting material [32]. The property of changing resistance can be utilized to detect concentrations of different analytes by correlating the changes in resistance to the concentration or pressure of a target species. Various interactions can lead to a change in resistance, such as adsorption, molecular binding, and changes with regard to the material itself [33]. Common conducting materials include but are not limited to metal oxides [34], conducting polymers [35], and carbon allotropes [36]. Chemiresistors can be used for either gas-phase [37] or liquid-phase sensing [38]. When using chemiresistive sensors, one must take into account the film thickness and the voltage. As the films get thicker, the size of the response to the analyte increases, and the film is more stable. However, the response time is much slower. With thinner films, the opposite effect is seen, with fast response times, less stable films, and smaller response sizes. For gas sensors, higher voltages can be used for thinner films to achieve larger responses. In liquids such as water, however, the voltage cannot be too high, since electrochemical side reactions or hydrolysis could occur. Functionalization of the conducting material, either through covalent or physical interactions, can be used to improve selectivity of the sensor to a certain analyte [39].

ChemFETs, on the other hand, utilize an indirect method of ion detection. For this method, the ions collected on the selective membrane apply an electric field perpendicular to the actual conductive material. This electric field changes the current going from the source through the gated channel to the drain of the device, which can be correlated to the concentration of ions. A positive electric field (generated by cations) attracts electrons and repels electron holes in the channel. A negative electric field (generated by anions) attracts electron holes and repels electrons in the channel. In a p-type device, cations decrease the conductivity of the channel and anions increase it, and vice versa for n-type devices [40]. These devices can be made selective by using an ion-selective membrane over the gate that modulates the channel, which generates an electric field as specific ions are captured [41].

These sensors are quite simple to fabricate and give good current responses for low concentrations of analyte. They also do not require a counter electrode or a reference electrode, simplifying the set-up greatly compared to other methods such as potentiometry. Due to its small size, it can also be used in situ. However, in complex sample matrices, pH and conductivity affect the sensor response, as well as non-specific binding (physisorption).

### 2.4. Electrochemical

#### 2.4.1. Potentiometry (Ion-Selective Electrodes, ISEs)

Potentiometry is the observation of changes in the electrochemical potential of an electrode in solution with respect to a reference potential in order to determine analyte concentration. This method requires both a working electrode and a reference electrode. The working electrode can either be a blank electrode for redox potential (ORP) measurements, or have a membrane selective to the desired analyte, while the reference electrode remains at a constant potential. The most commonly used reference electrodes are the saturated calomel electrode (SCE) and the silver/silver chloride (Ag/AgCl) electrode. For the blank electrode, the double layer formed on the surface causes a change in potential. As the conductance of a sample solution increases, shrinkage of the double layer is induced, which results in a change in potential with respect to the reference electrode [42]. For the ion-selective electrode, the membrane on the working electrode can have two possible modes of performance: redox equilibrium and ion capture. Redox equilibrium works through the redox interaction between the analyte and the membrane on the working electrode. The redox interaction between the analyte and the membrane either oxidizes or reduces the analyte, generating a potential difference as the reaction approaches equilibrium. Ion capture utilizes ion-selective membranes to trap analyte molecules onto the electrode. The concentration of ions on the electrode generates a potential difference [43]. The potential differences can be correlated to concentration using the Nernst equation (Equation (3)).

(3) $$E_{cell}=E_{cell}^{0}-\frac{RT}{nF}lnQ_{r}.$$

Using this equation, one can use the potential differences to obtain the concentrations in solution [44]. The method is quite sensitive, since the potential differences in the electrodes are normally in the hundred-millivolt range, which allows for readouts through inexpensive commercial voltmeters. 

Electrochemical methods have the advantage over chemiresistive sensors as they are invariant to changes in the bulk of the electrode, even though they are equally sensitive to changes in the surface chemistry. Their main drawback is the requirement for a reference electrode, which requires frequent maintenance and calibration. Similar to other solid-state sensors, they may be miniaturized for use in microfluidics. They can be used for continuous online monitoring, but are also subject to interferences by pH, conductivity, and other analyte species.

#### 2.4.2. Anodic Stripping Voltammetry (ASV)

Anodic stripping voltammetry (ASV) is a method that allows for preconcentration of the analyte to obtain a lower limit of detection. ASV utilizes three electrodes (as opposed to two in the case of potentiometry). These are the working electrode, the reference electrode, and a counter electrode. To prepare for analysis, the potential between the working and the counter electrode is kept higher than the oxidizing potential of the analyte to remove residual ions from the electrode. Then, the potential is lowered so that the analyte electroplates onto the electrode. Finally, the potential is slowly raised to oxidize the electroplated analyte and dissolve it back into solution. This provides the stripping current as a function of the oxidation potential. Oxidation releases electrons, which is measured as current [45]. The current given by the deposition can be correlated to the concentration through the Levich equation (Equation (4)), which gives the deposition current as a function of the electroplated analyte.

(4) $$i{\left(t\right)}_{dep}=0.62nFAD^{\frac{2}{3}}\omega^{\frac{1}{2}}\mu^{-\frac{1}{6}}C\left(t\right),$$

where *i* is the current, *n* is the number of electrons in the half reaction of the analyte, *F* is the Faraday constant, *A* is the active area, *D* is the diffusion coefficient, *ω* is the rate of stirring of sample solution, *μ* is the kinematic viscosity, and *C* is the analyte concentration. There are three different ways to strip the electroplated analyte from the working electrode: linear ramp stripping voltammetry, alternating current (AC) stripping voltammetry, and differential pulse stripping voltammetry (DPV). Linear ramp stripping increases the potential linearly as a function of time. This is sufficient for identifying different adsorbed species; however, there is a large non-Faradaic contribution to the current, which contributes to the noise. AC voltammetry is phase-sensitive, which means that it can separate the Faradaic current (current related to redox processes) from the non-Faradaic current. This is because the reversible processes occur within the timescale of the alternating potential. Irreversible processes are, therefore, eliminated [46]. DPV is similar to the linear ramp in that there is a linear potential increase. However, at fixed time interval, the potential pulses to a higher potential before returning to the linear ramp. This is good for small amounts of analyte at sub-ppb levels [47]. 

ASV is highly sensitive, and the stripping potential is different for each cation, meaning interference is minimized and simultaneous detection of analytes is possible. However, three electrodes are required for analysis (a working electrode, a counter electrode, and the reference), which complicates the set-up and results in the need for frequent maintenance and re-calibration.

### 2.5. Spectrometry

#### Inductively Coupled Plasma Mass Spectrometry (ICP-MS)

ICP-MS is a very common method for determination of metals in drinking water. This method allows for ppt levels of detection for many elements. Samples for ICP-MS are in the liquid phase and are incorporated into argon plasma through the use of a nebulizer. The electrically charged argon plasma (charged via induction heating) dissociates the molecules, and then ionizes them by removing one electron. These ions are then scanned using a quadrupole mass spectrometer, which separates the ions based on mass-to-charge ratio (*m*/*z*). It does this by setting its voltage and radio frequency to allow certain ions to pass through to the detector while all others are ejected. Copper(I) ions, for example, have a mass-to-charge ratio of 63/1. When a specific voltage and radio frequency are applied, only copper ions pass through. Since some molecules do not get fully atomized, there can be interferences if a molecular fragment is of the same mass as the analyte. To remove interferences, one can use either a collision cell or a reaction cell. The collision cell uses the fact that molecular fragments are bigger than elements and, therefore, undergo more collisions in an inert gas. This means that the molecular fragment loses its kinetic energy faster than the element, which retains most of its kinetic energy. This allows for the passage of elements, while stopping interfering molecular fragments from passing through to the detector. The reaction cell focuses on the thermodynamics of the interaction between an element or molecular fragment and a reactive gas. A molecular fragment reacts exothermally with a reactive gas, whereas an element reacts endothermally. This allows for passage of elements to the mass spectrometer while ejecting the molecular fragments. Both these methods increase the resolution and selectivity of ICP-MS [48].

This method is the most sensitive out of all of the methods since it is able to detect individual atoms as they are passed through the quadrupole mass analyzer. Since atoms have different masses, simultaneous detection is also possible using mass spectrometry. However, the method is destructive since it requires atomization of the sample. The method also requires large and very expensive instrumentation, making in situ measurements impossible.

## 3. Analytes

### 3.1. Barium

Barium is a soft metal used to make various things such as paint, bricks, ceramics, and tiles. It is also used as an additive for fuels, sealants, and the passivation of limestone. Barium occurs in water naturally through soil erosion and leeching of barium ore, but it can also occur through other means such as industrial emissions. In humans, high intake of barium can affect kidney function and promote cardiovascular disease. The World Health Organization has a guideline value (not a maximum limit) of 1.3 ppm. Health Canada has a higher proposed maximum allowable concentration (MAC) of 2 ppm [49,50].

A fluorescent method based on imination of an anthracene molecule for the detection of barium(II) was developed by Basa et al. This method uses a 1,2-phenylenediamine host with an anthraquinone macrocycle to improve selectivity toward barium(II) in solution. When tested with barium(II) titration in acetonitrile, a linear increase was seen in fluorescence intensity going from 0–133 µM (0 ppm to 18 ppm), after which the sensor molecule saturated. Although there are no other analytical data available, this sensor shows potential for the detection of barium(II) in aqueous media [51].

A potentiometric sensor based on dimethyl-4,4-dimethoxy-5,6,5’,6’-dimethylene dioxy biphenyl-2,2-dicarboxylate (DDB)—a liver drug—for the detection of barium(II) was developed by Hassan et al. This method consists of using DDB as the barium(II)-selective ionophore and coating it onto an Ag/AgCl internal reference electrode for use as the working electrode. An Ag/AgCl external electrode was used as the reference for this test. When testing this barium(II)-sensing method, a linear range of 10 µM to 0.1 M (1.4 ppm to 13 733 ppm) was found with a limit of detection of 5 µM (0.7 ppm). When tested against other cations for interference, barium(II) gave the highest slope for potential changes compared to other metal cations, making this method quite selective. In real samples, the values obtained by this method were in good agreement with those obtained by the standard AAS method [52].

A potentiometric method based on 3-deoxy-d-erythro-hexos-2-ulose bis (thiosemicarbazone) (DHUT) as the ionophore for detection of barium(II) was developed by Zamani et al. The electrode was prepared using a solution of DHUT in plasticizer and coating it onto an Ag/AgCl wire for use as the working electrode. An Ag/AgCl electrode was used as the external reference for this method. When tested for sensitivity to barium(II) by titration, the potential of the electrode changed proportionally to the amount of barium(II) added. A linear range of 1 µM to 0.01 M (0.1 ppm to 1373 ppm) was obtained. A limit of detection of 0.56 µM (77 ppb) was observed for this method. When testing against other cations, the response slope for barium(II) was much higher compared to the other cations, meaning that this method is sufficiently selective. In real samples, the values obtained were in good agreement with the standard AAS method [53].

An AAS method for detection of barium(II) in water was developed by Silva et al. This method uses a tungsten coil to atomize the samples for analysis by AAS. As the concentration of barium(II) changed in the solution, the peak height for the characteristic barium(II) wavelength would increase proportional to the amount of barium(II) in solution. Although a linear range is not available, a detection limit of 0.2 ppb was obtained, which is much lower than the limits set by the World Health Organization (WHO) and Health Canada. Interference studies with other cations showed that only calcium(II) interfered with the peak, but the interference could be corrected with addition of Ethylenediaminetetraacetic acid (EDTA). In real water samples, the values obtained with this method were in good agreement with the standard ICP-AES method used for comparison [54].

### 3.2. Cadmium

Cadmium is a heavy metal that is harmful to human health. It is classified as a carcinogen, and can have adverse effects on the kidneys, bones, and respiratory system. Cadmium occurs naturally in water through volcanic activity and erosion, but it also occurs through human activities such as mining, fossil fuel combustion, and recycling of electronic waste. Although cadmium levels in drinking water are usually low, the increase in these human activities may affect levels currently found. For water to be considered safe to drink, the World Health Organization recommends a maximum limit of 3 ppb, whereas Health Canada set a higher limit of 5 ppb [55,56].

A colorimetric method for the detection of cadmium(II) using gold nanoparticles (AuNPs) modified with 4-amino-3-hydrazino-5-mercapto-1,2,4-triazoles was developed by Wang et al. This sensor functions through the cadmium(II) chelation-induced aggregation of the functionalized AuNPs, which results in a change in color of the solution from red to blue. The addition of cadmium(II) into solution led to a shift in the absorbance peak from 520 nm to 650 nm, which was proportional to the concentration of cadmium(II) in solution. The ratiometric response obtained was correlated to the concentration added to the solution, and a linear range from 60 nM to 480 nM (7 ppb to 54 ppb) was obtained, with an *R*^2^ value of 0.9963. A limit of detection of 30 nM (3.5 ppb) was observed for this sensor. When comparing to other cations for interference, the ratiometric response given by the cadmium(II) chelation was twice as large as the response by other cations, making this a selective colorimetric method for the determination of cadmium(II) [57]. 

A napthalimide-based fluorescent sensor for determination of cadmium(II) was developed by Wang et al. This sensor utilizes an *N*,*N*′-bis(salicylidene)diethylenetriamine receptor to turn on fluorescence in the presence of cadmium(II) in solution. This sensor is pH-dependent; at low pH ranges (<4.5 pH), the free molecule fluoresces, and shows no enhancement of emission after interaction with cadmium(II). Within the pH range of 7.0 to 13.5, the free molecule does not fluoresce. However, upon addition of cadmium(II), fluorescence is enhanced in solution, which would make this a viable method in environmental conditions. In a 1:1 ethanol (EtOH)/H_2_O solution buffered with 4-(2-hydroxyethyl)-1-piperazineethanesulfonic acid (HEPES) buffer at pH 7.2, the fluorescence enhancement of the molecule at 525 nm was linearly proportional to the concentration of cadmium(II) in solution. A linear range of 50 nM–10 µM (6 ppb to 1 ppm) was obtained with an *R*^2^ value of 0.9902 and a limit of detection of 520 nM (58 ppb). When compared against other cations, only cadmium(II) led to a fluorescence enhancement, making this sensor quite selective [58].

A protein-based fluorescent sensor for cadmium(II) was developed by Varriale et al. This method utilizes a column packed with zinc(II)-saturated Chelex resin with a rhodamine-labeled metallothionein. Cadmium(II) was flowed through the column, displacing the rhodamine-labeled metallothionein, and leading to a fluorescence enhancement. When water with no metal ions was flowed through, there was no fluorescence detected in the fluorometer. Once cadmium(II) aqueous solution was flowed through, however, the metallothionein was eluted, resulting in a fluorescence enhancement at 575 nm, which was proportional to the concentration of cadmium(II) flowed through the column. Cadmium(II) solutions with a concentration range of 2.5 ppb to 10 ppm were flowed through the column, which resulted in a limit of detection of 0.5 µM (56 ppb). When testing against other cations, no other cations led to the elution of the metallothionein, meaning that this method is selective toward cadmium(II) [59].

A potentiometric method for the detection of cadmium(II) was developed by Ion et al. This method uses a cadmium(II)-specific ionophore to change the potential of the working electrode. Changes in the potential can be correlated to the concentration of cadmium(II) in solution. The ISE membrane containing the ionophore, *N*,*N*,*N*′,*N*′-tetradodecyl-3,6-dioxaoctanedithioamide (ETH 5435), was glued to a plasticized polyvinyl chloride (PVC) tubing with a PVC/tetrahydrofuran (THF) slurry. The reference used was an Ag/AgCl electrode. In a 0.1 mM sodium ion background at pH 7, cadmium(II) resulted in a change in potential proportional to its concentration in solution. A limit of detection of 11 ppt was obtained, which is much lower than the values set by the WHO and Health Canada [60].

A thermally enhanced ASV method for the determination of cadmium(II) was developed by Marken et al. This method utilizes the microwave radiation-enhanced deposition of cadmium(II) onto the working electrode, which enhances the stripping peak and the detection limit. A microwave working electrode with a 100-µm Pt disc was used as the working electrode, along with a Pt-mesh counter electrode and an SCE as the reference electrode. With no heating, there was no detectable stripping peak present for 400 µM (45 ppm) of cadmium(II) in 4.6 pH acetate buffer. When heated up to 205 °C, the peak was present at −0.603 V for the same concentration. The enhancement in the peak was proportional to the temperature going from 30–205 °C. Although there was no quantitative analysis in this study, this method shows potential for a highly sensitive cadmium(II) sensor [61].

**Figure 1:**
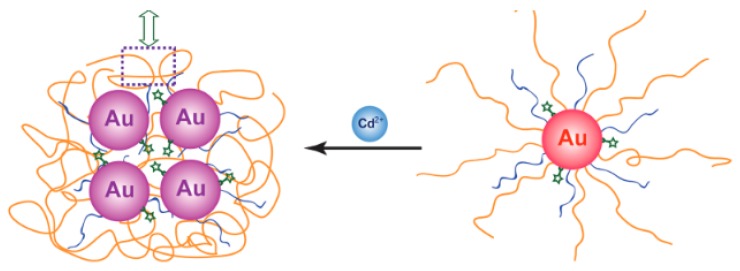
Mechanism of the cadmium(II)-induced aggregation of gold nanoparticles (AuNPs). Reprinted with permission from Reference [59]; Copyright 2011 American Chemical Society.

An SERS method for determination of cadmium(II) was developed by Yin et al. This method is dependent on Raman-active AuNPs tagged with a Raman-active dye, and a cadmium(II)-selective polymer. Upon chelation of cadmium(II) onto the nanoparticle, the AuNPs aggregate, which turns on the Raman signal and leads to a 90-fold enhancement of the Raman signal (Figure 1). 

When cadmium(II) was added to an aqueous solution of functionalized AuNPs, a Raman peak enhancement was seen at 525 cm^−1^, proportional to the amount of cadmium(II) added. A detection limit of 1 µM (112 ppb) was observed for this method. When testing against other cations, cadmium(II) gave the biggest Raman peak enhancement at 525 cm^−1^, with zinc(II) giving a much smaller response, and all other cations not enhancing the peak at all [62].

An alizarin-based SERS probe for detection of trace levels of cadmium(II) in drinking water was developed by Dasary et al. Alizarin was functionalized onto the AuNP as a Raman reporter, while 3-mercaptopropionic acid was used as a cadmium(II) chelating agent. Upon addition of cadmium(II), aggregation of AuNPs induced by the chelation of cadmium(II) onto the 3-mercaptopropionic acid leads to a Raman enhancement, which can be used to determine the concentration of cadmium(II) in water. In a pH 8.5 buffer, enhancement of the Raman peak at 1335 cm^−1^ was seen, which was proportional to the amount of cadmium(II) added into the solution. A linear range of 25 ppb to 200 ppb was obtained, with a saturation point at 250 ppb. The detection limit of this method was found to be 10 ppt, which is quite sensitive. Comparison of the cadmium(II) response to other cations showed that there is no Raman peak enhancement present with the other cations. When testing in real water samples by spiking cadmium(II), recovery was shown to be adequate, and the detection limit in environmental water was found to be 70 ppt [63].

An AAS method using preconcentration in a knotted reactor was developed by Wen et al. For this method, the solution of cadmium(II) was mixed with an ammonia solution, and then injected into the knotted reactor, where the now precipitated cadmium hydroxide was adsorbed onto the knotted reactor walls. Then, a 1 M nitric acid solution was passed through the reactor, eluting the cadmium hydroxide and moving it into the nebulizer for analysis by FAAS. This method gave a linear correlation for the absorbance of the cadmium peak with the concentration of cadmium(II) eluted from the reactor. The linear range obtained went from 40 ppt (the detection limit) to 2 ppb, with an *R*^2^ value of 0.999. Interference tests showed that there was no significant effect on recovery of cadmium(II) when in the presence of various different cations and anions. When tested in various certified reference and real samples, the values obtained by this method were in good agreement with the certified reference samples, and with the standard ICP-MS method of detection [64].

An AAS method for cadmium(II) detection through solidification of floating organic drop microextraction was developed by Dadfarnia et al. For this method, the sample containing cadmium(II) was mixed with 0.2 M iodide at pH 1.2. This solution was transferred into a solution of 0.02 M methyltrioctylammonium chloride in 1-undecanol. This was mixed until the CdI_4_^2−^ formed previously reacted with the methyltrioctylammonium chloride, and then precipitated in an ice bath. This precipitate was then melted and dissolved in ethanol for analysis. When using this method, a linear correlation between the absorbance and concentration of cadmium(II) was obtained, with a range of 80 ppt to 30 ppb, and an *R*^2^ value of 0.9998. A limit of detection of 8 ppt was observed, which makes this method highly sensitive. When compared against other cations and anions, the recovery of cadmium(II) was not significantly affected, making this method quite selective to cadmium(II). When this method was tested in real samples, recovery values were normally above 97%, and the values obtained were in good agreement with the standard graphite furnace (GF) AAS method used for comparison [65].

### 3.3. Chromium

Chromium is an element found in the Earth’s crust, most commonly in its trivalent state. Chromium, as an element and as its various salts, is used for tanning, pigments, photography, and alloy production. Most chromium occurring in the environment comes from human activity, such as refineries and thermal generating stations. Humans actually require 0.5–2.0 µg of chromium(III) daily. Chromium(III) does not have any harmful effects that are known, but chromium(VI) is a known carcinogen. The World Health Organization set a provisional guideline value of 50 ppb, which is the same as the maximum allowable concentration for Health Canada [66,67]. Since this review is focused on cation detection, only chromium(III) detection is discussed here, since chromium(VI) is usually found as a chromate anion.

**Figure 2:**
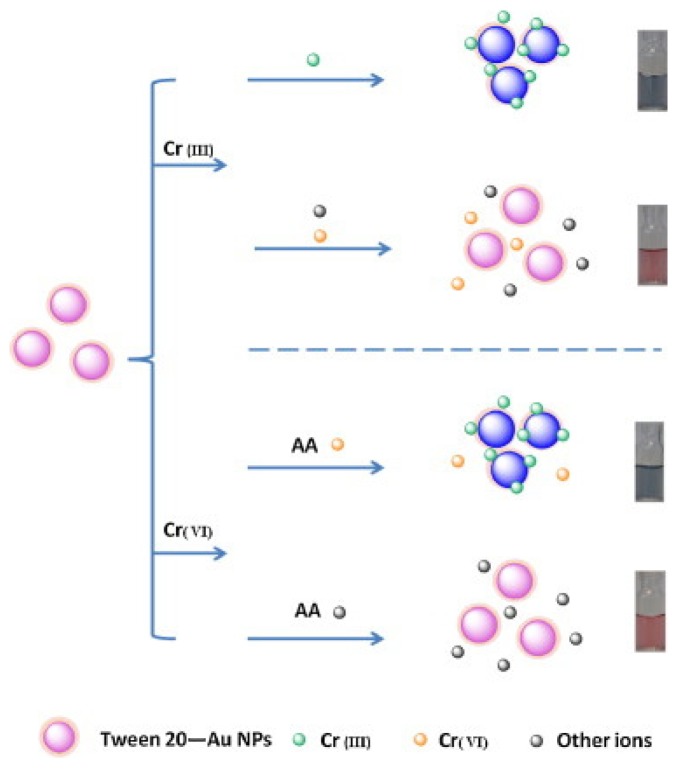
The sensing mechanism of the functionalized AuNPs. Reprinted with permission from Reference [65]; Copyright 2015 Elsevier.

A colorimetric method based on citrate chelation on Tween-20-stabilized AuNPs for the detection of chromium(III) was developed by Wang et al. In this method, the Tween-20-stabilized AuNPs functionalized with citrate are dispersed in a phosphate buffer solution. Upon interaction with chromium(III), the AuNPs aggregate, causing a visual color change from red to blue. This method only induces the color change when exposed to chromium(III) and not chromium(VI) (Figure 2).

When chromium(III) was tested with this method, the absorption peak at 520 nm decreased while the peak at 660 nm increased. This change in the absorbance spectrum was proportional to the concentration of chromium(III) ions in solution. The ratiometric response was correlated with the concentration of chromium(III), showing a linear range of 0.05–5.0 µM (3 ppb to 260 ppb), and an *R*^2^ value of 0.989. A detection limit of 0.016 µM (0.8 ppb) was obtained for this method. When tested with other cations, no other cations interfered with the signal or led to aggregation of the AuNPs, making this method quite selective. This method was also used for testing in real water samples using the sample addition method, and the recovery values remained above 91% [68].

**Figure 3:**
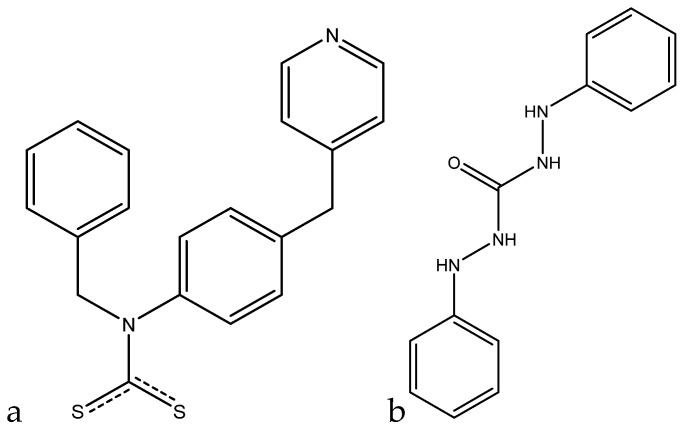
The structure of (**a**) the dithiocarbamate-modified *N*-benzyl-4-(pyridin-4-ylmethyl)aniline (BP-DTC) ligand and (**b**) 1,5-diphenylcarbazide.

A colorimetric method that utilizes dithiocarbamate-modified *N*-benzyl-4-(pyridin-4-ylmethyl)aniline ligand (BP-DTC)-functionalized AuNPs for the detection of chromium(III) was developed by Zhao et al. This method, much like the one before, also works through the aggregation of AuNPs via chelation of chromium(III) to the BP-DTC ligand (Figure 3a).

When exposed to chromium(III) in aqueous solution, a color change from red to blue is visually observed. In the absorbance spectrum, the absorbance peak at 520 nm decreases upon addition of chromium(III), and a new peak at 630 nm arises, with the peak heights being proportional to the amount of chromium(III) added. A linear range for the ratiometric response was found between 0 and 8.0 µM (0 ppb and 416 ppb) with an *R*^2^ value of 0.9958. A limit of detection of 31 ppb was obtained for this method. When testing against other cations, the response of the chromium(III) was five times larger than any of the other interfering cations, making this method quite selective. When tested with real water samples, the values obtained were in good agreement with the standard ICP-MS method, and sample addition gave recovery values above 103% [69].

A fluorescent method for the detection of chromium(III) through the use of a distyryl boron-dipyrromethene (BODIPY) derivative was developed by Wang et al. For this method, the BODIPY derivative by itself does not have an emission peak due to the amine groups transferring electrons to the BODIPY backbone, quenching fluorescence. Once chromium(III) is in solution, the electrons from the amine groups now transfer to the chromium(III) metal center, turning on the fluorescence for the molecule. When this molecule is exposed to chromium(III) in aqueous acetonitrile solution, an emission peak at 643 nm arises, and the peak height is proportional to the chromium(III) in solution. Although no analytical data are available, the sensor had a quantitative fluorescence enhancement from 0–200 µM (0 ppb to 10 ppm), indicating that this sensor has a large range. When tested against other cations for interference, the BODIPY derivative only bound to chromium(III) and did not give any fluorescence enhancement with other cations. However, iron(III), copper(II), and mercury(II) did affect the ability of chromium(III) to enhance fluorescence (although concentrations of 10 ppm were required) [70].

A potentiometric method based on carbon nanotube (CNT) coated Pt electrodes for the detection of chromium(III) was developed by Abbaspour et al. In this method, an multi-walled (MW) CNT/PVC membrane is used as the working electrode, with 1,5-diphenylcarbazide as the chromium(III)-selective ionophore, and an SCE as the reference electrode (Figure 3b). When this sensor was tested with chromium(III) in solution, a pH range of 3–7 was found to be optimal, since higher pH values lead to chromium hydroxide being formed, while lower pH values lead to protonation of the ionophore. The potential changes with respect to the SCE reference were correlated with the concentration of chromium(III) in solution, and a Nernstian response for the concentration range of 30 nM to 0.01 M (1.5 ppb to 520 ppm) was found, with a limit of detection of 32 nM (1.6 ppb). When tested against other cations, chromium(III) displayed better Nernstian responses than any of the other interferences tested. In real samples, the values obtained by this method were in good agreement with the standard AAS method [71].

An SERS method of detecting chromium(III) through the use of citrate-functionalized AuNPs was developed by Ye et al. This method utilizes the chromium(III)-induced aggregation of the functionalized AuNPs to enhance the Raman signal generated by the bare AuNPs. When tested with chromium(III) in aqueous solution, the Raman peak at 555 cm^−1^ saw an enhancement proportional to the amount of chromium(III) added. A calibration curve was obtained with a range of 50 nM to 0.2 µM (2.6 ppb to 10 ppb), with an *R*^2^ value of 0.936. The limit of detection for this method is 50 nM (2.6 ppb). When tested against other cations, only chromium(III) led to a visual color change form red to blue and an enhancement in the Raman spectra, making this method quite selective. When tested in real water samples using the sample addition method, recovery values were all above 94% [72].

An SERS method for detection of chromium(III) using silver nanoparticles (AgNPs) was developed by Liang et al. This method utilizes AgNPs along with lateral flow immunoassays to quench the Raman peak intensity proportional to the concentration of chromium(III). When the sample solution of functionalized AgNPs was flowed through the immunoassay, they were bound by the antigen Cr-EDTA-bis(trimethylsilyl)acetamide (BSA). When a mixture of chromium(III) solution and AgNPs was flowed through, the chromium(III) prevented the AgNPs from being bound by the antigen, decreasing the Raman peak signal. When tested with chromium(III), a decrease in the characteristic peak at 1077 cm^−1^ is seen, which is proportional to the concentration of chromium(III) in solution. A linear range of 0.01 ppt to 0.01 ppb was seen, with an *R*^2^ value of 0.99774. A limit of detection of 0.01 ppt was obtained. When tested against other cations, only chromium(III) led to the quenching of the Raman peak at 1077 cm^−1^, making this method quite selective. In real water samples, however, the values were much lower than what was obtained by ICP-MS, meaning that there is still some optimization required for this method [73].

An AAS method with preconcentration of a packed minicolumn for the determination of chromium(III) was developed by Rao et al. This method uses flow injection to adsorb the chromium(III) in solution to the C_18_-packed minicolumn. Here, 5 ppm manganese(II) is added to the chromium(III) solution to enhance the absorbance signal, and the pH is adjusted to 7. This solution is then flowed through the minicolumn for preconcentration. The chromium(III) is then eluted with methanol and flowed into the nebulizer for analysis with FAAS. When this method was tested with chromium(III), a linear range was obtained for 20 ppt to 200 ppb. The limit of detection obtained was 20 ppt. When tested with other cations, there was no effect on the recovery of the chromium(III) from the minicolumn. In real samples, the value obtained by this method was in good agreement with the certified value for the sample [74].

### 3.4. Copper

Copper is an essential element for human life. The recommended intake for adults is 900 μg/day, and, for children, it is between 340 and 890 μg/day. Deficiency in copper intake can lead to various adverse effects, such as anemia and low white blood cell count [75]. This is not a concern, however, since food intake supplies enough copper for a human. Overexposure to copper can lead to gastrointestinal issues (nausea, diarrhea, etc.). High levels of copper in water also stain clothing and plumbing. In Canada, drinking water has a maximum allowable concentration of 2 ppm copper, with an aesthetic objective of 1 ppm [76].

**Figure 4:**
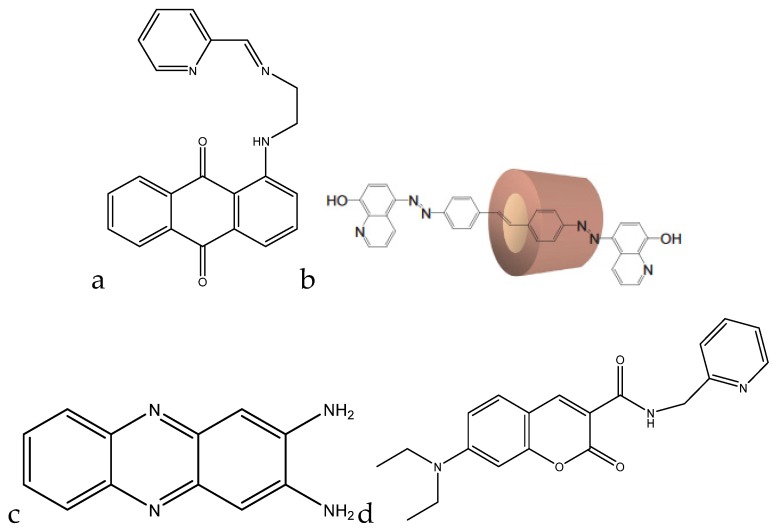
Structure of (**a**) the copper(II)-selective anthraquinone derivative, (**b**) rotaxane with 8-hydroxyquinoline as the blocking group (reprinted with permission from Reference [75]; Copyright 2010 Elsevier), (**c**) 2,3-diaminophenazine, and (**d**) coumarin-based fluorophore.

Kaur et al. developed a colorimetric sensor based on 1-aminoanthracene-9,10-dione-based molecules for the detection of copper cations (Figure 4a). 

When copper(II) was added to the chemosensor in a methanol (MeOH)/H_2_O (1:1) mixture at pH 7.0 (10 mM HEPES), the λ_max_ shifted from 494 nm to 604 nm, which was accompanied by a color change from red to blue. Below pH 5, the chemosensor protonates and does not form a complex with copper(II). Between pH 5 and 8.75, a complex is formed, with the complex formation reaching completion around pH 7. Above pH 8.75, the complex separates into Cu(OH)_2_ and the free chemosensor. This chemosensor also had a ratiometric response to copper(II). When copper(II) was titrated against the chemosensor, a decrease in the peak at 494 nm was seen, accompanied by a proportional increase in the peak at 604 nm. A calibration curve was able to be made, correlating the ratio of the peak heights to the added concentration of copper(II), which ranged from 5–150 μM (0.318–9.533 ppm) [77]. 

Park et al. developed a colorimetric detection method using a rotaxane dye (Figure 4b). The rotaxane coupled with the hydroxyquinoline allowed for a 43% enhancement in absorbance. At a pH of 7.5 in dimethylformanilide (DMF)/H_2_O (80:20, wt.%), the λ_max_ shifted from 440 nm to 520 nm. The color change was also able to be detected down to 170 ppb, with the upper limit being dependent on the concentration of the dye. Since the color change was also ratiometric (similar to the previous chemosensor), a calibration curve can be formed with this dye as well, allowing for interpolation of concentration values in unknown samples [78].

Udhayakumari et al. utilized 2,3-diaminophenazine for fluorometric detection of copper(II). With the addition of 100 μL of a 1.5 × 10^−5^ M Cu^2+^ solution in water to 3 mL of 5 × 10^−5^ M 2,3-diaminophenazine in acetonitrile, a quenching in fluorescence was observed, with the emission peak at 500 nm decreasing in intensity with the addition of the copper(II) (Figure 4c). The sensing is feasible at a pH range of 4–8. Below pH 4, the amine groups protonate, and the complex is unable to form. Above pH 8, the complex dissociates to form the sensor molecule and Cu(OH)_2_. The molecule is reported to have a detection limit of 0.015 ppb and was shown to be selective to copper(II) when tested against various interfering cations [79].

A coumarin-based fluorogenic probe was developed by Jung et al. for determination of copper(II) in living cells (Figure 4d). The excitation peak of the fluorophore is located at ~480 nm in an aqueous HEPES/dimethylsulfoxide (DMSO) (9:1, *v*/*v*) solution. Once copper(II) is added, the fluorescence is nearly fully quenched. The quenching of the fluorescence is also proportional to the amount of copper(II) present in solution, with a negative linear correlation with respect to the emission intensity. The linear range of the sensor goes from 0.5 μM to 50 μM (32 ppb to 3.2 ppm) [80].

An ASV method for determining trace amounts of copper(II) was developed by Zhao et al. This method involves using a carbon nanotube (CNT) thread as the working electrode, a platinum wire as the counter electrode, and an Ag/AgCl reference electrode. CNT thread was used as the working electrode since it has high conductivity, good mechanical strength, and a large surface area. The copper(II) was deposited using a deposition potential of −1.5 V. Osteryoung square wave stripping was used for stripping the metals from the CNT thread. When copper from the working electrode is oxidized to copper(II), a peak in the current-voltage (I–V) curve appears at +24 mV. The current peak at this potential is proportional to the concentration of the copper(II) in solution, with a linear response from 0.5–3.5 μM (32 ppb to 222 ppb, *R*^2^ = 0.99). The effects of dissolved oxygen on the response of copper(II) were also investigated. When oxygen is present in solution, the copper(II) stripping peak moves to −56 mV, and the peak becomes sharper and more pronounced. The slope of the calibration curve also increases, going from 0.33 to 0.60 from a range of 1.5–5.0 μM (96 ppb to 320 ppb). Since the copper(II) stripping potential is quite specific to copper(II), this is a very sensitive method for copper(II) detection in water [81].

**Figure 5:**
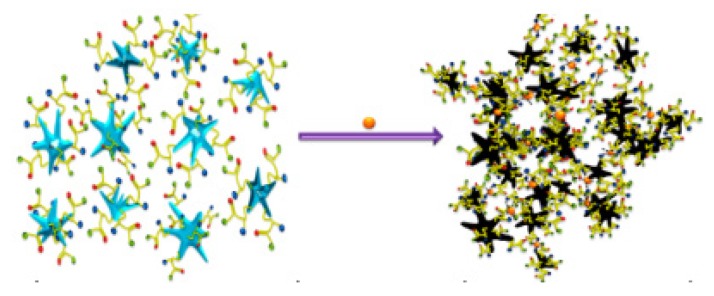
Complexation induced aggregation of cysteine-functionalized gold nanostars (Cys-AuNSs). Reprinted with permission from Reference [79]; Copyright 2014 American Chemical Society.

SERS was utilized to detect copper in aqueous media by Ndokoye et al. through the use of cysteine-functionalized gold nanostars (Cys-AuNSs). When the Cys-AuNSs are adsorbed onto a colloidal gold surface, the signals from 1500–900 cm^−1^ are enhanced, and the symmetric vibration mode of COO is observed strongly at 1400 cm^−1^. A 2:1 Cys-AuNS:Cu^2+^ complex is formed, which causes the aggregation of the AuNSs (Figure 5). 

This greatly enhances the SERS signal due to plasmon coupling. When tested with other metals, they complexed with the Cys-AuNS, but did not induce the aggregation, which indicated selectivity to copper(II). The method was tested over a range of 8.5–40 μM (544 ppb to 2.56 ppm) and saw a proportional increase in the symmetric vibration mode of the COO group at 1400 cm^−1^ [82].

A flame atomization AAS (FAAS) method was developed by Cassella et al. This method utilizes a flow injection method to preconcentrate a minicolumn, and then elutes the copper(II) from the column for analysis by FAAS. The sample, which is maintained at pH 9, is pumped into the minicolumn packed with a polystyrene/divinylbenzene resin functionalized with (*S*)-2-[hydroxyl-bis-(4-vinyl-phenyl)-methyl]-pyrrolidine-1-carboxylic acid ethyl ester, which chelates to copper(II). Here, 2 M HCl was then flowed through the minicolumn to desorb the copper(II) from the resin for analysis by FAAS, which measured at a wavelength of 324.8 nm. Absorbance values were measured for concentrations between 10 ppb and 200 ppb, and the increases in absorbance were linear with respect to copper(II) concentration (*R*^2^ = 0.9995). Environmental samples were also tested and spiked to determine recovery, and they were found to be in agreement with the standardized method, electrothermal AAS (ET AAS). Recoveries were also quite good, ranging from 91% to 106% (only sea water samples had a low recovery value of 79.5% compared to the rest of the samples) [83].

An online concentration determination method based on AAS was developed by Porento et al. Aqueous copper samples were injected via syringe pump into the nitrogen plasma jet for atomization, and then were flowed into the AAS for analysis. For a range of concentrations from 0.4 to 3.9 ppm, a linear correlation was observed between copper(II) concentration and the absorbance at 324.8 nm, with an *R*^2^ value of 0.99. The limit of detection for this method was 0.25 ppm. Response was also compared against magnesium(II), which did not give any discernible response at 324.8 nm [84].

### 3.5. Hardness

Water hardness is the amount of combined calcium and magnesium (and various other divalent cations at lower concentrations) in water. The WHO defines it as the capacity of water to react with soap, i.e., the harder the water is, the more soap it needs to form a lather. The most common expression for hardness is the concentration of calcium carbonate per liter. The hardness of water is quantified as follows: <60 ppm is soft, 60–120 ppm is moderately hard, 120–180 ppm is hard, and >180 ppm is very hard [85]. The most common sources of ions that contribute to water hardness are from sedimentary rock (limestone and chalk) and soil runoff. Both calcium and magnesium are essential for human biology. Calcium increases bone mass and reduces the risk of fracture. Calcium deficiency can increase the chances of osteoporosis, hypertension, stroke, and various other cardiovascular issues. On the other hand, an excess of calcium can lead to hypercalcemia in those who are prone to milk alkali syndrome [86]. Magnesium is a cofactor for 350 cellular enzymes and is involved in protein and DNA/RNA synthesis. Magnesium deficiency can lead to hypertension, while excessive intake can have a laxative effect [87]. Although there is no strict guideline, hardness levels between 80 ppm and 100 ppm are recommended [88].

A method of determining water hardness through the use of acoustic wave sensors was developed by Veríssimo et al. A 9-MHz quartz crystal coated with an ionophore solution (1,3,5-tris [10(1-adamantyl)-7,9-dioxo-6,10-diazaundecyl] benzene (Mg ionophore), 10,19-bis[bis(octadecylcarbamoyl) methoxyacetyl]-1,4,7,13,16-pentaoxa-10,19-diaza cycloheneicosane (Ca ionophore), polyvinyl chloride (PVC), plasticizer, and lipophilic salt in 5 mL THF) was used to deposit the calcium(II) or magnesium(II) onto the coated quartz crystals. Calibration solutions were flowed through the quartz crystal cells through flow injection analysis to create a calibration curve to interpolate real sample values. The change in frequency of the quartz crystal was correlated to the calibration concentration that was injected. By doing this, linear calibrations for both calcium(II) and magnesium(II) were obtained, with *R*^2^ values of 0.9990 and 0.9994, respectively. These calibration curves were then used to analyze real water samples in Portugal, and the results were compared to the standard EDTA titration method. Both methods were in agreement with each other, with no discrepancies being present between the two [89].

An optical test strip for the determination of water hardness was developed by Capitán-Vallvey et al. This test strip utilizes an ion exchange mechanism which quantifies calcium(II) and magnesium(II) simultaneously. The strips were prepared on Mylar, upon which the 4,13-[bis(*N*-adamantylcarbamoyl)acetyl]-1,7,10,16-tetraoxa-4,13-diazacyclooctadecane (K22B5 ionophore) in THF was spin-coated. The strip was then fully submerged into the sample solution for 5 min. The strip was then removed, and its absorbance was measured against a background of Mylar. By using equimolar solutions of calcium(II) and magnesium(II), a calibration curve was made using calibration solutions, and was fit to a theoretical response function using the logarithm of the concentration as the independent variable. A detection limit of 1.9 ppm was obtained, with a linear range of 1.9 ppm to 14 800 ppm. This method was also used in real samples and was compared to a standard complexometric titration. The results obtained with the test strips were quite comparable to the values obtained by the titration, making this an easy method to sense water hardness [90].

A complexometric method of determining water hardness was developed by Bhattacharjee et al. The method is based on the properties of EDTA titration. A channel is three-dimensionally (3D) printed from acrylonitrile butadiene styrene (ABS). It contains a red and blue light emitting diode (LED), with a photodiode for each. An inlet and outlet were present to inject the sample. A calgamite solution was prepared at pH 10 as the indicator. The sample was then injected into the device. Afterward, the calgamite solution was injected, changing the color to red, and dropping the voltage output by the blue LED. Once EDTA-Na_2_ was added, the solution turned blue, and the voltage output from the blue LED increased. By measuring the absorbance of the blue light emitted, a calibration curve was created with a linear range of 0 ppm to 120 ppm, and an *R*^2^ value of 0.9163. Although the sensor is not very precise, it can be used to differentiate between soft and hard water at the 60-ppm threshold [91].

A hardness determination method based on fluorescence resonance energy transfer (FRET) was developed by Dey et al. For the energy transfer, two dyes, acriflavine (Acf) and rhodamine B (RhB), are used as the energy donor and acceptor, respectively. FRET efficiency (efficiency of energy transfer) can be affected by metal ions in water, such as calcium(II) and magnesium(II), and the changes in efficiency can be correlated to water hardness. It is seen that, when Acf and RhB are both in solution with calcium(II) and magnesium(II), the efficiency of the FRET decreases from 11.37% to 4.38%. The changes in FRET efficiency were observed from 30 ppm to 200 ppm in clay dispersion, although it was not a linear correlation; thus, a calibration curve could not be created. A FRET efficiency of 48.2% was determined as the threshold above which water would be considered soft, and below which water would be considered hard [92].

**Figure 6:**
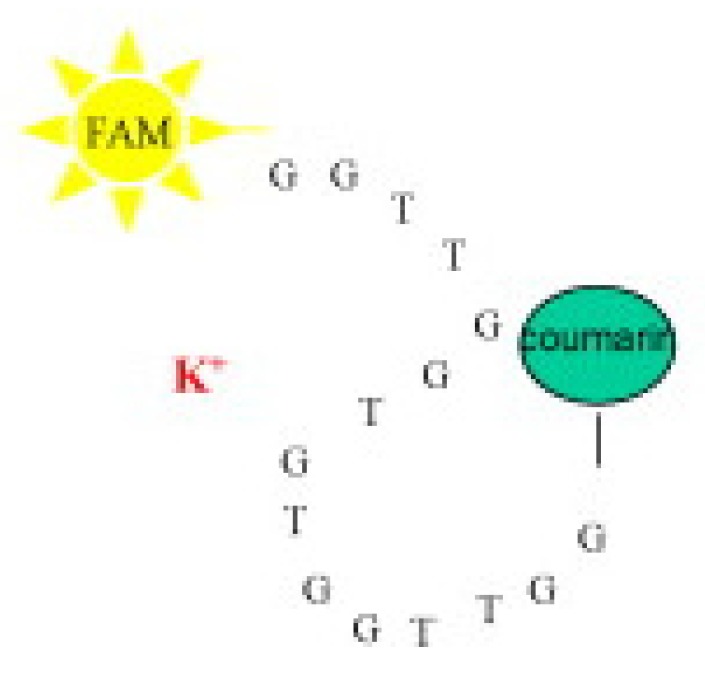
The molecular aptamer beacon used for detection. Reprinted with permission from Reference [90]; Copyright 2008 Elsevier.

A fluorometric method of hardness determination using a molecular aptamer beacon was developed by Lerga et al. (Figure 6). 

For the analysis, a calibration curve was created ranging from 0–3000 μM (0 ppm to 300 ppm) in a 0.15-mL solution containing 100 nM beacon in 10 mM HEPES (pH 8.4). The concentration of the calcium(II) and magnesium(II) was plotted against the quenching of the fluorescence at 518 nm, and a calibration curve was created with an *R*^2^ value of 0.998. Real samples were also tested, and the method was compared with AAS. The values obtained with the beacon were quite comparable to those obtained with AAS, with maximum variation being in the range of 0.5 mM (50 ppm) [93].

A method of hardness determination using a potentiometric sensor array was developed by Saurina et al. This method used a working electrode with a selective ionophore mixture (calcium ionophore II, ammonium ionophore I, potassium ionophore III, sodium ionophore III, lithium ionophore VI, magnesium ionophore I, and hydrogen ion ionophore III) to detect calcium(II) and magnesium(II) in solution. To obtain the calibration curve, different volumes of the standard ion solution were added to a 0.01 M Tris solution, and the changes in potential were correlated with the concentration. For calcium(II), this resulted in a dynamic range of 20 μM to 300 μM (2 ppm to 30 ppm), with a limit of detection (LOD) of 0.006 mEq/L (milliequivalents per liter, 120 ppb). For magnesium(II), the range was from 2 mM to 10 mM (200 ppm to 1000 ppm) with an LOD of 1.7 mEq/L (21 ppm). Although the magnesium LOD is higher than what is recommended, this method is good for testing the general hardness of a water sample [94].

**Figure 7:**
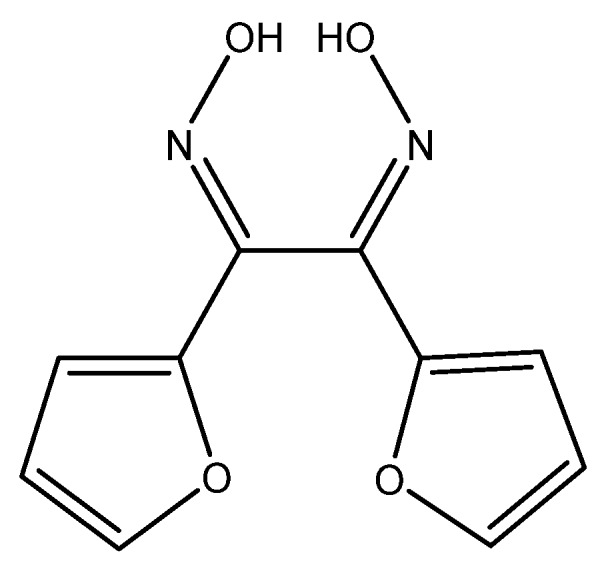
Structure of α-furildioxime.

A PVC-based membrane sensor for water hardness was developed by Singh et al. This sensor utilizes α-furildioxime as a neutral carrier for a calcium(II)-selective electrode (Figure 7). 

Various parameters for the construction of the ion-selective membrane were investigated, such as the type of plasticizer and the ratios of each component. The optimal result was obtained by using α-furildioxime/PVC/dibutylphthalate/potassium(tetrakis-4-chlorophenyl)borate (KTpClPB) in a 4:32:62:2 (wt.%) solution in THF. By running the calibration solutions to obtain a curve, a linear range of 2.56 × 10^−7^ M to 1 M (26 ppb to 100,000 ppm) was observed, as well as an LOD of 1.25 × 10^−7^ M (13 ppb). This range is stable between pH 3.5 and 9.0, which would indicate that, below pH 3.5, the ionophore begins to protonate, and, above pH 9, the calcium(II) forms its hydroxide Ca(OH)_2_. When measuring real samples, the calcium(II)-selective electrode obtained similar concentrations to those obtained by AAS [95].

### 3.6. Lead

Lead enters drinking water when lead-containing service pipes begin to corrode. Since lead has the ability to bioaccumulate in the body overtime, there is no safe maximum concentration. For practical purposes, a maximum allowable concentration of 5 ppb was set by Health Canada [96]. Infants and young children are the most susceptible to lead poisoning due to the effects on their physical and mental development. Lead exposure in children is linked to growth defects, nerve damage, and decreased function of blood cells. In adults, lead exposure can lead to adverse cardiovascular effects, impaired kidney function, and reproductive problems [97].

**Figure 8:**
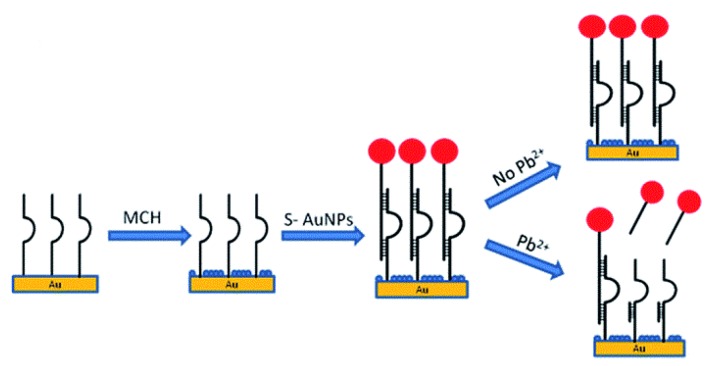
Sensing mechanism of the lead(II)-selective quartz crystal microbalance (QCM) method. Reprinted with permission from Reference [95]; Copyright 2014 Royal Society of Chemistry.

A DNAzyme-based QCM-D method for measurement of lead(II) ions was developed by Teh et al. This sensor uses a 5-MHz gold-coated quartz crystal as the microbalance. The crystal was firstly functionalized with a thiol-modified GR-5 strand, then filled with 6-mercaptohexanol, a blocking agent. An AuNP-hybridized DNAzyme was then introduced to bind with the immobilized GR-5. Upon the addition of lead(II), the bound AuNP/DNAzyme is released, which increases the frequency and decreases the dissipation factor of the QCM. This can be correlated to the concentration of lead(II) in solution (Figure 8).

The increases in frequency were correlated to the concentrations of lead(II), and a linear relationship was obtained for the range of 46–3000 nM (10 ppb to 622 ppb) with an *R*^2^ value of 0.997. The detection limit of the sensor was determined to be 14 nM (3 ppb). When the dissipation was correlated with the concentration, a range of 66–3000 nM (14 ppb to 622 ppb) and a detection limit of 20 nM (4 ppb) were observed (*R*^2^ = 0.994). When tested against other interferences, the DNAzyme was only released in the presence of lead(II), making it quite selective. Tap-water samples were also tested and gave comparable values to the standard ICP-MS method [98].

A colorimetric method for lead(II) detection using polyazomacrocycles was developed by Ranyuk et al. When using a triamide-substituted diaminoanthraquinine-linked polyazomacrocycle, a blue shift was seen in the presence of lead(II) from 571 nm to 524 nm (in a 50 μM solution of HEPES buffer, pH 7.4). This blue shift accompanies a visual change of the solution from violet to pink. This change is quantitative, as different concentrations of lead(II) in solution proportionally blue-shift the absorbance peak. Through UV/vis titration, the detection limit was determined to be 21 ppb, with a molar extinction coefficient of 4.9 × 10^3^ L∙mol^−1^∙cm^−1^. When tested against silver(I) and cobalt(II), no distinct changes were observed, showing that this sensor is selective to lead(II) [99].

**Figure 9:**
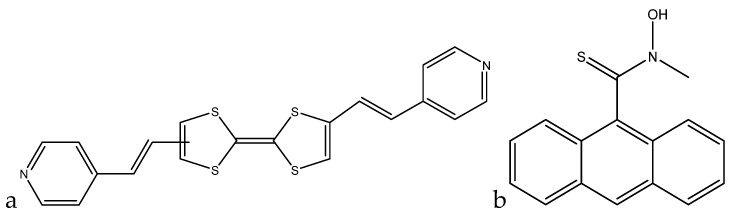
The **(a**) TTF-π-pyridine derivative used for lead(II) sensing and (**b**) the fluorescent anthracene derivative for lead(II) detection.

A colorimetric method of lead(II) detection based on tetrathiafulvalene (TTF)-π-pyridine derivatives was developed by Xue et al. The interaction between the pyridyl groups and lead(II) leads to the color change seen in the molecule solution (Figure 9a).

The absorbance peaks at 301 nm and 440 nm decrease proportionally with the increase of the new peaks at 330 nm and 55 nm with increasing concentrations of lead(II). This is accompanied by a visual color change in the solution from yellow to purple. A linear range for this sensor was obtained going from 0–6.1 × 10^−5^ M (0 ppb to 13 ppm) [100].

A fluorescence method based on anthracene derivatives for the detection of lead(II) was developed by Chae et al. This molecule is complexometric, and binding to lead(II) enhances fluorescence in an aqueous solution (Figure 9b). Prior to exposure to lead(II), the sensor molecule possesses weak fluorescence at 420 nm due to the lone pairs on the thioamide group. When lead(II) complexes at the thioamide group, fluorescence is enhanced due to the lone pairs being used for complexation. The fluorescence is only linearly proportional up to 0.5 equivalents of lead(II), which indicates that the sensor molecule binds to the lead(II) in a 2:1 fashion. Although analytical data are not available for this sensor molecule, it shows promise as a quantitative fluorometric method to measure lead(II) in aqueous solution [101].

A method of fluorescent lead detection through the use of catechin-synthesized Au nanoparticles was developed by Wu et al. This sensor is based on the lead(II)/catechin complexes and the lead/gold alloy that forms on the catechin/AuNP surface, mimicking the catalytic activity of the hydrogen peroxide oxidation of Amplex UltraRed. This enhances the emission peak at 588 nm when excited with a 540-nm source. This enhancement is linearly proportional to the concentration of lead(II) ranging from 10 nm to 10 μM (2 ppb to 2 ppm, *R*^2^ = 0.99). A limit of detection of 1.5 nM (0.3 ppb) was achieved for this method. When compared to other cations, only lead(II) gave the fluorescence enhancement at 588 nm, indicating the selectivity of the probe. When comparing the method to AAS for real sample analysis, a *t*-test indicated that the results were not significantly different [102].

The ASV method used by Ruecha et al. mentioned for zinc(II) detection was also used for lead(II) detection. By using square wave ASV, the stripping current was correlated to the lead(II) concentration in solution at a stripping voltage of −0.75 V. A linear range of 1 ppb to 300 ppb was achieved for the method, as well as a detection limit of 0.1 ppb. In human serum, detection of lead(II) using the sample addition method gave recovery values close to 100%, suggesting that this method is quite accurate for detection of lead(II) [103].

The ASV method of copper(II) detection used by Zhao et al. was also used for lead(II) detection. The lead(II) was deposited at −1.5 V, and it exhibited a sharp stripping peak at −0.488 V. A linear correlation between the stripping current and the concentration of lead(II) was obtained over the range of 1.0–4.0 μM (207 ppb to 829 ppb) with an *R*^2^ of 0.99. The calculated limit of detection was 1.5 nM (0.3 ppb) [81].

A method of lead(II) detection using a gold nanoparticle/reduced graphene oxide (AuNP/rGO) colloid for SERS was developed by Zhao et al. This sensor utilizes lead(II)-enhanced gold leaching. This reduced the amount of AuNPs on the rGO, which decreased the SERS intensity of the rGO. These decreases in the Raman intensity at 1350 cm^−1^ were correlated to the concentration of lead(II). A linear range of 5–4000 nM (1 ppb to 829 ppb) was obtained with an *R*^2^ value of 0.9926. The limit of detection obtained for this method was 1 nM (0.2 ppb). When testing in real samples, the sample addition method provided recoveries above 90%. When testing against an array of other cations, only lead(II) was able to induce gold leaching on the AuNP/rGO surface, which means that this method is quite selective to lead(II) [104].

An SERS method of detecting lead(II) based on a DNAzyme was developed by Wang et al. This method takes advantage of the catalytic reaction that occurs when the DNAzyme binds to lead(II). Onto a gold surface, the DNAzyme was immobilized. A substrate-modified gold nanoconjugate was bound to the substrate. When lead(II) was present, the bond between the substrate and the DNAzyme was cleaved, decreasing the intensity in the Raman signal. The decrease in the peak at 1584 cm^−1^ was correlated to the concentration of the lead(II), which gave a detection range of 20 nM to 1 μM (4 ppb to 207 ppb), with a detection limit of 4 ppb. When tested against other cations for interference, there was no significant decrease in the signal (although some of the cations, such as zinc(II), are known to cleave the substrate–DNAzyme bond), indicating that this method is selective [105].

A flow injection method for the determination of lead(II) through FAAS was developed by Rodriguez et al. This method uses a packed microcolumn to preconcentrate the lead(II), which is then eluted and run through the nebulizer, using AAS for analysis. The microcolumn is packed with silica gel treated with a mixture of Aliquot 336 and nitroso-R-salt. When testing the effects of pH on the adsorption of the lead(II) by preconcentrating between pH 3 and 7, an optimal range of pH 5.2 to 5.9 was obtained; thus, the tests were carried out at pH 5.5. Different eluents (HCl, HClO_4_, and EDTA) were also tested for removal of lead(II) from the column, and HCl was found to be the best eluent, since EDTA was inefficient in the removal of lead(II), and HClO_4_ removed the nitroso-R-salt from the column. Using the 217-nm resonance line, a calibration curve was obtained over the range of 0 ppb to 100 ppb, and was found to have a linear correlation, with a limit of detection of 4 ppb. When tested against various other cations and anions for interference, only nickel(II) and fluoride caused any interference. However, since the concentrations of these ions in water are negligible, there is no need to take any extra precaution. When tested with real water samples, the values obtained were in line with the values obtained by the standard ET AAS method, with no significant difference at 95% confidence [106].

An FAAS method for the determination of lead(II) using a microcolumn was developed by Ensafi et al. This method, just like the one previously described, utilizes a microcolumn to preconcentrate the lead from solution. However, rather than silica gel, this microcolumn is packed with activated carbon loaded with pyrogallol red. Pyrogallol red has a high binding constant with lead(II) between pH 5 and 6.5, and, since pyrogallol red is a polycyclic and aromatic, it can adsorb onto the activated carbon through π–π stacking interactions. To elute the lead(II) from the column, a 0.5 M solution of HNO_3_ was used, since higher concentrations did not improve recovery from the column. When testing a wide array of cations, no other cation was observed to interfere, since pyrogallol red is quite specific to lead(II). When testing real samples using the sample addition method, recoveries between 97% and 104% were obtained, showing that this method is quite reliable for sensing [107].

### 3.7. Mercury

The main source of mercury in water is through atmospheric deposition or the discharge of industrial wastes. In water, mercury, usually in the form of methylmercury, is bioconcentrated by fish. Health Canada recommends an MAC of 1 ppb for mercury, due to the fact that long-term exposure leads to adverse neurological effects. Large doses can cause irreversible damage to the central nervous system. Dermal exposure can cause toxic dermatitis and eczema [108].

Rasheed et al. developed three rhodamine-based methods for the colorimetric detection of mercury(II) in aqueous solution. The first two methods are based on the fluorescence enhancement of rhodamine B derivatives from complexation with mercury(II) in solution. The first rhodamine B derivative (TS) uses a mercury(II)-selective 2-aminothiazole receptor with the rhodamine backbone as the fluorophore. When tested in acetonitrile/water (7:3 *v*/*v*, 10 mM HEPES at pH 7.0) solution, an enhancement was seen in both the absorbance and the fluorescence spectra at 580 nm and 559 nm, respectively. This was accompanied by a visual color change from colorless to pink upon complexation (due to the complexation-induced spirolactam ring opening). The limit of detection for this sensor is 0.326 µM (65 ppb), and the linear range goes up to 12 µM (2 ppm). The second rhodamine B derivative (PST) replaces the 2-amiinothiazole receptor with a 2-amino-5-bromopyridine receptor. When tested in acetonitrile/water (8:2 *v*/*v*), the same absorbance and fluorescence peaks were observed at 580 nm and 559 nm, respectively. This was also accompanied by a visual change in color from colorless to pink. The limit of detection for this derivative is 0.63 µM (126 ppb), and the linear range goes up to 10 µM (2 ppm). When both derivatives were tested against other ions for interference, none gave the colorimetric response that mercury(II) exhibited, making these sensors quite selective [109,110]. The third method is based on the functionalization of an alternating copolymer vesicle using a rhodamine B derivative for the detection of mercury(II). The previously mentioned derivative PST was immobilized onto the self-assembled copolymer vesicle through the nucleophilic substitution of the bromide on the pyridine ring. When tested in aqueous solution, the same absorbance and fluorescence peaks were seen at 580 nm and 559 nm, which was accompanied by the same color change from colorless to pink. The limit of detection for this method, however, is much lower at 53 nM (10 ppb), with the linear range going up to 10 µM (2 ppm) (*R^2^* = 0.998) [111].

**Figure 10:**
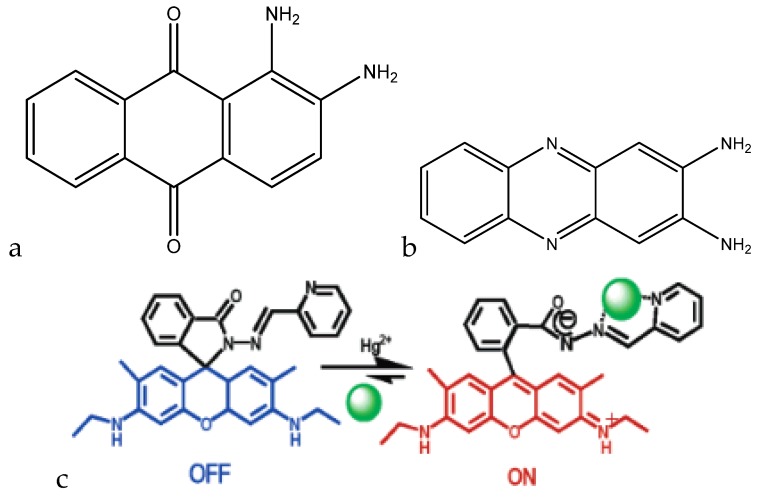
The structures of (**a**) 1,2-diaminoanthraquinone and (**b**) 2,3-diaminophenazine, and (**c**) the detection mechanism of the rhodamine 6G fluorophore (reprinted with permission from Reference [109]; Copyright 2007 American Chemical Society).

A ratiometric method of detecting mercury(II) was developed by Kim et al. This method uses the change in the absorbance spectrum induced by the complexation of mercury(II) to deduce the concentration of mercury(II) in a sample (Figure 10a). 

Upon the titration of mercury(II) into a 10^−4^ M solution of 1,2-diaminoanthraquinone in DMSO, the absorbance peak at 528 nm decreases proportionally to the amount of mercury(II) added. In addition, an increase in the absorbance peak at 461 nm is seen. When compared to other cations, none gave the change in the absorbance spectrum that mercury(II) gave, which shows the selectivity of this sensor molecule. Although there are no analytical data with regard to this molecule, it shows promise as a selective mercury(II) colorimetric sensor [112].

A method of colorimetric detection of mercury(II) using silver nanoparticles (AgNP) was developed by Firdaus et al. This method takes advantage of the fact that mercury(II) has a higher reduction potential than silver(I). Thus, the AgNPs ionize in the presence of mercury(II), changing the color of the solution from yellow-brown (due to aggregation of AgNPs) to colorless. Upon addition of mercury(II) to the sensor molecule, a decrease in the absorbance peak at 420 nm was observed, which was proportional to the amount of mercury(II) added. A linear range of 5 μM to 300 μM (1 ppm to 60 ppm) was obtained with an *R*^2^ value of 0.9988, and a limit of detection of 0.85 μM (170 ppb) was achieved. When comparing to other interfering cations, only the mercury(II) gave a significant decrease in absorbance at 420 nm, making this a very selective molecule for mercury(II) detection (although the LOD is too high for public use). Real sample tests using the sample addition method gave recovery values of above 95%, which demonstrates the selectivity of the molecule in a matrix full of interfering ions [113].

A fluorescent method of detecting mercury(II) through the use of 2,3-diaminophenazine (DAP) particles was developed by Liu et al. The nanoparticles were fabricated through the UV irradiation of *o*-phenylenediamine (oPD) in aqueous solution. This irradiation leads to a yellow dispersion, which has a distinct emission wavelength (Figure 10b). DAP emits at a wavelength of 554 nm, which is quenched by the presence of mercury(II) in solution. When the mercury(II) was titrated into solution, the fluorescence intensity at 554 nm decreased proportionally to the concentration of mercury(II) over the range of 1 nm to 500 μM (0.2 ppb to 100 ppm), with a detection limit of 1 nM (0.2 ppb). When testing other cations for interferences, no other interfering cation gave the fluorescence quenching that mercury(II) did, which shows that the DAP nanoparticles are selective toward mercury(II) [114].

A rhodamine 6G-based fluorophore for the detection of mercury(II) was developed by Wu et al. This probe is complexometric and exhibits a fluorescence enhancement upon binding to mercury(II) (Figure 10c). Unbound, the fluorophore is found in the spirolactam phase, making it colorless in DMF/H_2_O media. Upon the complexation of mercury(II), the spirolactam ring opens, inducing fluorescence. When mercury(II) was titrated into a solution of the fluorophore, proportional emission enhancements were seen at 560 nm. A linear range was obtained going from 2 ppb to 20 ppb, with a limit of detection of 2 ppb. When comparing the response of mercury(II) to other cations for interference, only mercury(II) complexation led to ring opening, which provides enhanced fluorescence. This demonstrates the selectivity of the fluorophore for use as a mercury(II) sensor [115].

A potentiometric method using calixarene ionophores for mercury(II) detection was developed by Tyagi et al. The working electrode is made of a *p*-*tert*-butyl-calix[4]arenethioether derivative along with PVC and a sodium tetraphenylborate (NaTPB) anion excluder in THF, and an SCE is used as the reference electrode. Since the ionophore has a high binding constant with mercury(II), the potential changes due to the sulfur donor atoms in the calixarene ionophore complexing with mercury(II). This causes an increase in the potential, which can be correlated to the concentration of mercury(II) in solution. A linear range was obtained going from 72 nM to 1 mM (14 ppb to 201 ppm). The sensor was not physically tested with other interferences; rather, ab initio calculations were used to determine the interactions. Soft metals show the strongest interaction, of which mercury(II) is the strongest [116].

An ASV method using AuNP/CNT composites for the detection of mercury(II) was developed by Xu et al. This method used a glassy carbon electrode modified with the AuNP/CNT nanocomposites to increase the number of electroactive sites for mercury(II) detection. Differential pulse (DP) ASV was used to strip the electroplated mercury from the electrode. A distinct stripping peak at +0.63 V is seen, which corresponds to the oxidation of mercury(II) from the electrode. This peak was proportional to the amount of mercury(II) in the solution, and it was correlated to the concentration to create a calibration curve. This calibration curve gave a linear range of 0.5 nM to 1.25 μM (0.1 ppb to 251 ppb) with an *R*^2^ value of 0.99. The detection limit of this method was determined to be 0.3 nM (0.06 ppb), which is much lower than the maximum allowable concentration of many countries [117].

**Figure 11:**
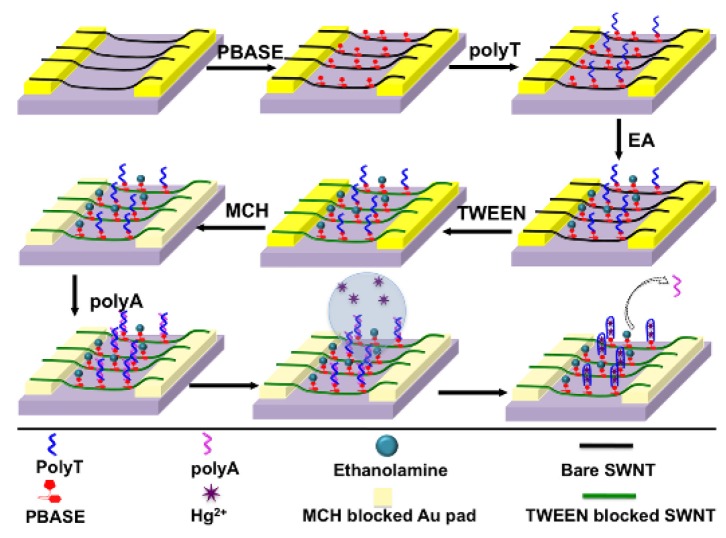
Schematic of the fabrication and sensing mechanism. Reprinted with permission from Reference [18]; Copyright 2013 AIP Publishing.

A chemiresistive sensor based on single-walled (SW) CNTs and structure-switching DNA for the detection of mercury(II) was developed by Gong et al. This method is based on the adsorption of amino-labeled polyT onto a SWCNT, followed by hybridization with polyA (Figure 11).

Once mercury(II) is exposed to the sensor surface, the polyT/polyA duplex is dehybridized, which releases the polyA from the surface and increases the conductivity of the sensor. The changes in conductivity were correlated to the concentration of mercury(II), and a linear correlation was found for the range going from 100 nM to 1 μM (20 ppb to 201 ppb). When tested against other cations, mercury(II) gave the largest change in conductivity of the film, having more than double the response, which proves the selectivity of this method [118]. 

An SERS method using gold nanostar dimers for mercury(II) detection was developed by Ma et al. This method functions through the mercury(II)-induced conjunction of the thymine base pair, which dimerizes the DNA-functionalized gold nanostars and enhances the Raman peak. A 60-nm gold nanostar dimer was chosen as the substrate for this experiment, and 4-aminothiophenol was used as the Raman reporter molecule. Upon addition of mercury(II), the 4-aminothiophenol peak at 1083 cm^−1^ was enhanced proportionally to the amount of mercury(II) added, due to the effect of mercury(II) on the dimerization of the gold nanostars. A linear range of 2 ppt to 1 ppb was obtained, with an *R*^2^ value of 0.99. The limit of detection obtained was 0.8 ppt, demonstrating the incredible sensitivity of this method. When comparing to other cations for interference, mercury(II) gave a Raman peak enhancement five times greater than the other cations, which shows that this method is selective as well [119].

A SERS method based on gold microshells for the detection of mercury(II) was developed by Han et al. This method uses an oligonucleotide-modified gold microshell, which undergoes a conformational change through exposure to mercury(II) ions. This conformational change occurs due to the mercury(II) ion being able to selectively bind to the thymine bases of two strands to form stable base pairs. This generates an enhancement of the Raman peak. Tetramethylrhodamine was used as the SERS signal generator for this method. With the addition of mercury(II), enhancement of the Raman peak at 1650 cm^−1^ is seen, which is proportional to the amount of mercury(II) added. A detection range was obtained going from 50 nM to 10 μM (10 ppb to 2 ppm), and, when the response was compared against other cations for interference, mercury(II) gave the highest enhancement even when the concentration of mercury(II) was five times less [120].

An AAS method based on solid-phase extraction to detect mercury(II) was developed by Pourreza et al. This method utilizes a minicolumn packed with 2-mercaptobenzimidazole and agar–agar. The sample is preconcentrated into the column, then eluted through the use of 3 M HCl, and analyzed via cold vapour (CV) AAS. Citrate buffer at pH 2.5 was found to be the optimal condition for the sorption of mercury(II) onto the column. The maximum absorbance signal was also obtained when the reducing agent concentration (concentration of SnCl_2_) was at 2.0%. A linear range for this method was obtained going from 40 ppt to 2.4 ppb, with an *R*^2^ value of 0.9994. The limit of detection for this method was also determined to be 20 ppt, much lower than limits set by many countries. When interference studies were performed, no other ion (except for Cl^−^ due to the formation of HgCl_4_^2−^) had any significant response. Real water samples were also tested using the sample addition method, and the recovery for each trial was above 95% [121].

### 3.8. Nickel

Nickel occurs in drinking water through the leaching of metals in the fittings found in household piping. The boiling of water in electric kettles can also introduce nickel into drinking water, specifically in decalcified kettles. Common adverse effects of nickel overconsumption include gastrointestinal issues, dermatitis, and oral hyposensitization. Based on the effects of nickel on the digestive system, a maximum limit of 70 ppb is recommended by the World Health Organization [122].

**Figure 12:**
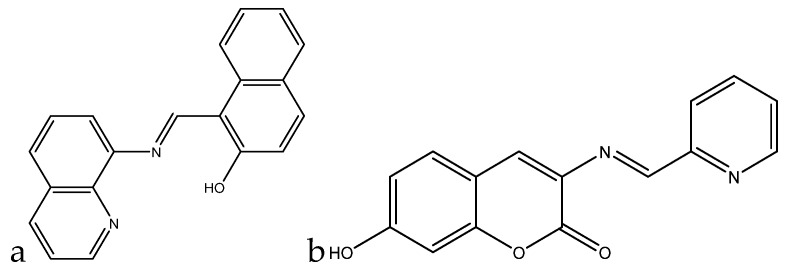
The (**a**) quinolone-based nickel(II) chemosensor and (**b**) the coumarin derivative used for nickel(II) detection.

A colorimetric method for the determination of nickel(II) in aqueous solution was developed by Liu et al. This method utilizes a quinolone based chemosensor, which undergoes a color change from yellow to red upon exposure to nickel(II) (Figure 12a). 

When tested in DMSO/H_2_O (1:1 *v*/*v*) in HEPES buffer at pH 7.4, the absorbance at 525 nm increased, following a linear correlation with an *R*^2^ value of 0.9887. A limit of detection of 0.22 μM (13 ppb) was obtained with a linear range of 8.6 μM to 15.2 μM (505 ppb to 892 ppb). When tested against other cations for interference, only nickel(II) was able to induce the yellow-to-red color change along with the increase in absorbance at 525 nm. Test strips were also fabricated with this chemosensor, with a limit of detection of 5.0 μM [123].

A coumarin-based colorimetric sensor for nickel(II) ions was developed by Jiang et al. This molecule experiences a red shift from 341 nm to 540 nm upon interaction with nickel(II), which is accompanied by a visual color change going from colorless to pink (Figure 12b). When tested with nickel(II) in EtOH/H_2_O (1:1 *v*/*v*), a ratiometric response was observed, with a simultaneous decrease in absorbance at 341 nm and an increase in absorbance at 540 nm. A detection limit of 0.5 μM (29 ppb) was obtained, and a linear range from 4 μM to 20 μM (235 ppb to 1 ppm) was observed with an *R*^2^ value of 0.995. When tested against other cations for interference, copper(II) and mercury(II) did induce red shifts of 50 nm and 84 nm, respectively. However, the red shift induced by nickel(II), as well as the enhancement in absorption, was much larger, making this molecule quite selective to nickel(II) [124].

A potentiometric method for the determination of nickel(II) was developed by Tomar et al. The nickel(II)-specific ionophore used was the novel Schiff base 3-aminoacetophenonesemicarbazone (AASC). To construct the nickel(II) ion-selective electrode (ISE), AASC, PVC, plasticizer (dibutylphosphate, DBP), and anion additive (sodium tetraphenyl borate, NaTPB) were combined in a ratio of 4:30:64:2 *w*/*w* in 5 mL of THF. This mixture was evaporated until an oily mixture remained. A Pyrex tube was coated with this mixture, and conditioned for 24 h by soaking it in a 0.01 M solution of Ni(NO_3_)_2_, while using a saturated calomel electrode (SCE) as an internal reference. When tested with nickel(II) in aqueous solution, a Nernstian slope was obtained for a range spanning 0.1 μM to 0.01 M (6 ppb to 587 ppb), with a detection limit of 51 nM (3 ppb). When tested against other cations for interference, nickel(II) gave the largest Nernstian slope, making this electrode quite selective to nickel(II). The electrode is stable between pH 2.0 and 9.8. Below a pH of 2.0, the ionophore begins protonating, preventing selective binding of nickel(II). Above pH 9.8, Ni(OH)_2_ formation occurs, preventing binding to the electrode. This electrode was also used in real samples (milk powder and chocolate), and the values obtained by the ISE were comparable to values obtained by the standard AAS method of detection, making this a reliable method for nickel(II) detection [125].

An ASV method for the detection of nickel ions using boron-doped diamond electrodes was developed by Musyarofah et al. The experiment was performed in 0.1 M phosphate buffer solution, using Pt wire as the counter electrode and an Ag/AgCl electrode as the reference. Deposition of the nickel(II) ions onto the electrode was performed at a potential of −100 mV for 5 min. When the potential was increased to strip the nickel from the electrode, a peak was seen at +1.1 V, with the current at that potential correlating to the concentration of nickel(II) in solution. A linear calibration curve going from 5 mM to 200 mM (293 ppm to 11,739 ppm) was obtained, with an *R*^2^ value of 0.9929. Although this method of detection is not very sensitive with respect to regulation, it shows potential as an electrochemical method for determination of nickel(II) in aqueous media [126].

An AAS method of detecting nickel(II) by chemical vapor generation in situ was developed by Matusiewicz et al. The sample is firstly vaporized by continuous flow hydride generation (using NaBH_4_ as the hydride donor). The vaporized nickel hydride is then trapped in an integrated atom trap and heated by an air–acetylene flame. Using this method, the detection limit was determined to be 0.21 ppb, with a linear range of 1 ppb to 50 ppb (*R^2^* = 0.9922). When tested with certified NIST (National Institute of Standards and Technology) samples, the values obtained by the AAS method were comparable to the certified values provided. Real samples were also tested and provided good recovery, making this a highly sensitive method for detection of nickel(II) in aqueous solutions [127].

### 3.9. Silver

Silver is commonly used as a disinfectant for commercial water filters. Leaching of the silver ions from the metallic silver used in the filter can introduce silver ions into drinking water sources. Although there is no evidence of silver having any adverse effect on human physiology (aside from argyria, which causes a blue discoloration of the skin), since it is not an essential element, any exposure to silver is unwanted. There is no strict guideline for silver in drinking water; however, the World Health Organization recommends an upper limit of 0.1 ppm [128].

**Figure 13:**
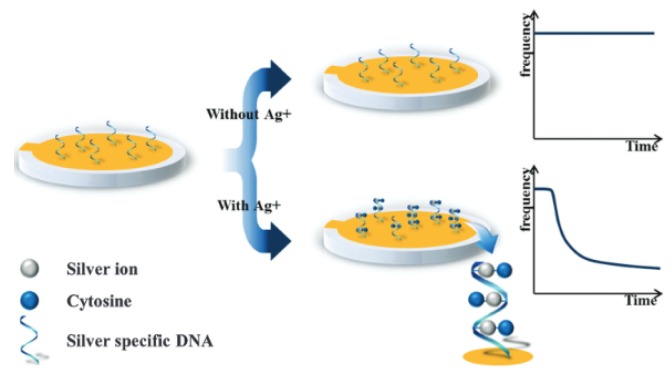
Illustration of the detection mechanism of silver(I). Reprinted with permission from Reference [117]; Copyright 2015 Royal Society of Chemistry.

Lee et al. developed a QCM method for detecting silver(I) in situ. For this, they use a quartz crystal functionalized with 5′–CCCCCCCCCCCCCCCCCCCCCCCCCCCCCC-3ThioMC–3′, a silver-specific nucleotide. For detection, a silver(I) containing solution was combined with a 1 mM solution of cytosine in a 1000:1 ratio. Calibration solutions ranged from 10 pM to 1 μM (0.001 ppb to 108 ppb) (Figure 13).

The limit of detection for this sensor is 100 pM (0.01 ppb), with a limit of quantification (LOQ) of 1 nM (0.1 ppb). However, when reusability was tested, a decrease in the change in frequency was observed (repeated exposure to 1 μM gave lower frequency shifts each time); thus, the sensor can only be used twice. Selectivity was also tested for this sensor by conducting the same experiment with various ions in the solution matrix. Only silver(I) was able to give a discernible signal at 1 μM (other ions were negligible). The sensor was also tested in real drinking water, where it maintained the same LOD and LOQ, although the frequency shifts were smaller [129].

A direct colorimetric detection method in aqueous solution for silver(I) was developed by Qin et al. This method utilizes a water-soluble organometallic polyelectrolyte which goes from colorless to yellow upon exposure to silver(I) ions. Upon the addition of 10 μM silver(I) (1.08 ppm), a red shift of 25 nm was seen at the λ_max_ of 390 nm. A calibration curve was made, correlating the red shift in the λ_max_ of the silver(I)-specific molecule to the logarithm of the concentration. A linear correlation was found between the two, with an *R*^2^ value of 0.99 (using 5 × 10^−6^ M sensor molecule). The linear range was determined to be 1 μM to 4 mM (108 ppb to 432 ppm) with a limit of detection of 0.5 μM (54 ppb) [130].

A detection method using gold nanoparticles for the colorimetric detection of silver(I) was developed by Lin et al. Gold nanoparticles are capped with citrate ions, which are then functionalized with Tween-20. Silver(I) is reduced and plated onto the surface of the gold nanoparticles, which causes Tween-20 to be removed from the gold nanoparticle, inducing aggregation. In the absorbance spectrum, this is seen as a drop in absorbance at 520 nm, and an increase in absorbance at 650 nm. The ratio of the absorbance was correlated to the concentration of silver(I) in solution. A calibration curve was made going from 0–1000 nM (0 ppb to 108 ppb), and it gave a linear range of 4 × 10^−7^ to 1 × 10^−6^ M (43 ppb to 108 ppb), with an *R*^2^ of 0.9935 [131].

**Figure 14:**
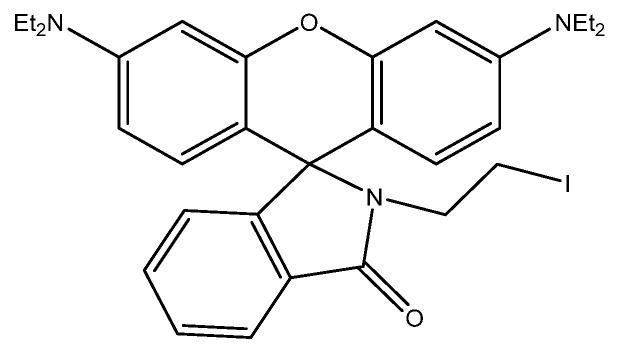
Structure of the rhodamine B derivative.

A fluorogenic probe for detecting silver(I) ions in water was developed by Chatterjee et al. The molecule used for detection, a rhodamine B derivative, is colorless in a 20% ethanol solution. Upon the addition of silver(I) ions, the solution turns pink, and a fluorescence peak arises at 584 nm. This occurs due to the spirolactam ring opening of the rhodamine B derivative induced by the silver(I) ions (Figure 14).

When tested against other cations, only the silver(I) led to ring opening of the molecule and induced a fluorescence enhancement. The fluorescence enhancement was linear with respect to the concentration of silver(I) over the range of 11 ppb to 540 ppb, and a limit of detection of 14 ppb was obtained [132].

**Figure 15:**
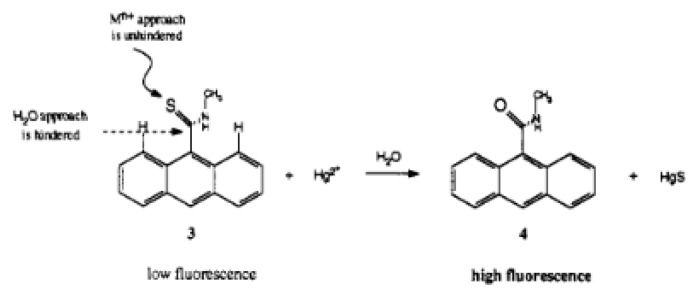
Mechanism of the oxidation of the thioamide group using mercury(II) as a reference. Reprinted with permission from Reference [121]; Copyright 1992 American Chemical Society.

Chae et al. developed a fluorometric method for determination of silver(I) using an anthracene derivative. This anthracene derivative utilizes the oxidation of a thioamide group to enhance fluorescence of the analyte solution proportionally to the concentration of the analyte (Figure 15).

When silver(I) was titrated into a 23 μM solution of the fluorescent molecule in 0.01 M 4-(2-hydroxyethyl)-1-piperazineethanesulfonic acid (HEPES) buffer (pH 7), a fluorescence enhancement was seen that was proportional to the silver(I) present in solution. The enhancement was linear up to two equivalents, indicating that this enhancement occurs in a 2:1 stoichiometry for silver(I) [133].

Polymer electrodes for the potentiometric detection of silver(I) in solution were developed by Rubinova et al. The selective membrane consists of *o*-xylylenebis(*N*,*N*-diisobutyldithiocarbamate) (copper(II) ionophore (I)), lipophilic cation exchanger, tetradodecylammonium tetrakis(4-chlorophenyl)borate (ETH 500), and methylmethacrylate–decylmethacrylate (MMA–DMA)/polyvinyl chloride/bis(2-ethylhexyl) sebacate (PVC/DOS) in a 9:1 ratio in THF. In methylene chloride solution, the PVC/DOS was omitted. Both solvents were used to determine which would be best for coating the gold wire for use in small volumes. The electrode was constructed by taking a gold wire and soldering it to a copper wire for electrical contact. The gold wire was then cleaned with sulfuric acid, then rinsed with acetone and left in chloroform for 3 min. A solution of poly(3-octylthiophene) was then added to the gold wire until the color of the wire was black. The membrane was then added by coating the wire with the membrane solution, and then evaporating the THF (or methylene chloride). The membrane made using methylene chloride solution was found to be best for microelectrode fabrication, and it was used for the experiment. A sodium-selective liquid-contact electrode was used for reference. Using this method, the electrode made in methylene chloride solution gave a detection limit of 0.63 nM (65 ppt), with a Nernstian response over the range of 1 nM to 10 μM (0.1 ppb to 1 ppm). These results were reproducible, and alternating concentrations showed that the response is reversible [134].

A calixarene-based ion-selective electrode for potentiometric detection of silver(I) was developed by O’Connor et al. The set-up was a conventional electrode utilizing a PVC membrane with various calixarene derivatives incorporated (with different ratios of sulfur and nitrogen groups). Using an SCE reference electrode, a detection limit of 100 μM (10 ppm), with a linear response slope of 51.74 mV/decade up to 0.01 M (1079 ppm), was obtained when the membrane was deposited onto glassy carbon electrode. When tested against various interferences, with the main interferences being sodium, lead(II), and mercury(II), mercury(II) and lead(II) poisoned the electrodes, with the silver(I) response decreasing after exposure to these ions (due to these ions being thiophilic). Sodium did not poison the electrode, however, and its presence did not affect the silver(I) response [135].

A square wave ASV method to determine silver(I) ions in surface water was developed by Schildkraut et al. This method utilizes a carbon paste electrode as the working electrode, with an SCE being used as a reference. Carbon paste was chosen over glassy carbon electrode and platinum electrode due to better sensitivity and less interference from the background. Water hardness was seen to have an effect on the stripping peak of the silver(I) at +0.170 V, which was seen through the decrease of the full width-half maximum (FWHM) as the hardness increased. Over the range of 0.2 ppb to 2 ppb, a linear correlation between peak current and silver(I) concentration in potassium hydrogen phthalate (KHP) buffer was found, with an *R*^2^ value of 0.991. Calibration curves were also made for different matrices, such as an NIST 1643c trace metal standard, a moderately hard synthetic water solution, and an SLRS-3 (St. Lawrence river) water reference. The calibration curves in the SLRS-3 and the moderately hard water were comparable to the original calibration curve in KHP buffer, but the calibration curve for the NIST 1643c had a much lower slope. This was found to be due to the higher acidity of the NIST 1643c (0 pH) compared to the SLRS-3 (1.6 pH). The NIST 1643c sample also splits the peak into two, with a smaller stripping peak appearing at +0.040 V. Due to this, a calibration curve correlating peak charge to concentration was created, which gave better slopes and higher correlation coefficients than the current measurements (*R*^2^ = 0.993). The detection limit of this method was 0.2 ppb [136].

An ASV method using polythiophene-modified platinum electrodes was developed by Zejli et al. The platinum thiophene electrode was placed in a stirred solution of 0.2 M KNO_3_ at pH 5 along with a platinum counter electrode and an Ag/AgCl reference electrode, and silver(I) was pipetted into the solution. The silver(I) was preconcentrated onto the electrode at a potential of −0.500 V, and the stripping current was scanned from +0.0 to +0.700 V. The characteristic silver(I) stripping peak was seen at +0.170 V. The pH of the solution was seen to have an effect on the stripping current seen, with the current increasing from pH 2 to pH 5 (the maximum), and then decreasing above pH 5 (possibly due to the formation of AgOH). A linear correlation of the current with respect to the silver(I) concentration was seen from 70 ppb to 1 ppm, with an *R*^2^ value of 0.995. The detection limit for this method was found to be 60 ppb. When testing various interferences, their stripping peaks were well separated from that of silver(I), which indicates that this method is quite selective [137].

**Figure 16:**
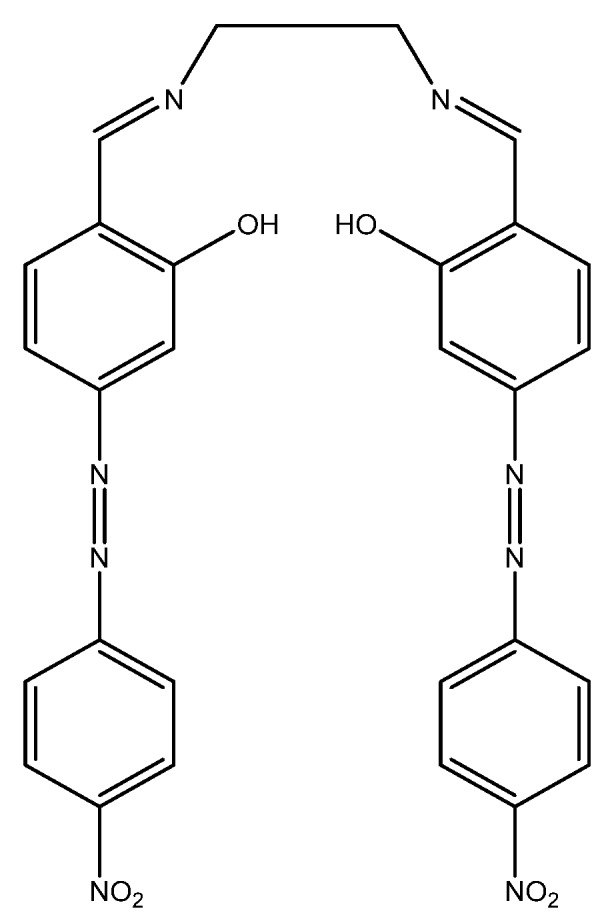
The structure of BNAS.

BNAS, a new Schiff base ligand, was used in an AAS method for the detection of silver(I) ions developed by Shamspur et al. This method utilizes a C_18_-bonded silica membrane that BNAS adsorbs onto to preconcentrate the membrane with silver(I) ions. The silver(I) is then eluted, and the concentration of silver(I) in solution is determined by FAAS (Figure 16). 

A 0.5 M thiosulfate solution was used to elute the silver(I) ion, and the optimal pH for extraction was found to be between pH 5 and 7.5. A linear range was obtained from 1 ppb to 50 ppb, with a limit of detection of 10 ppt. Interferences were also investigated to observe their effects on the recovery of silver(I). Although the interferences were in the ppm range, the recovery of silver(I) ions remained above 90% [138].

**Figure 17:**
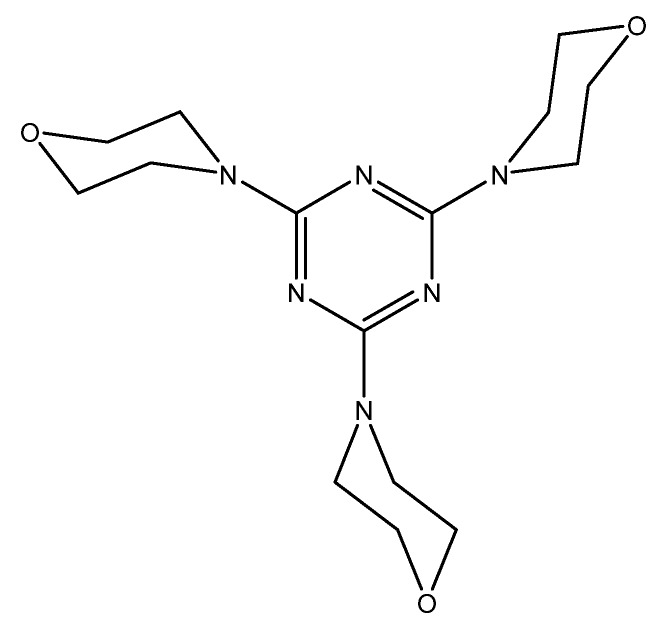
The structure of 2,4,6-trimorpholino-1,3,5-triazin.

An AAS method using a silica gel modified with 2,4,6-trimorpholino-1,3,5-triazin was developed by Madrakian et al. This method utilizes preconcentration in a packed column followed by elution and determination of the silver(I) concentration by FAAS at 328.1 nm (Figure 17).

The optimal pH range for adsorption onto the column was found to be pH 3–6, since the molecule protonates below pH 3 and silver(I) hydrolyzes above pH 6. For the experiment, the pH was kept at 3.5. Using 0.05 M thiosulfate for elution of solvent, a linear correlation was seen over the range of 125 ppb to 2.25 ppm. Interference studies also showed that there was no strong interference affecting the signal, and recovery of silver(I) remained above 94% [139].

### 3.10. Uranium

In the environment, uranium is commonly found in its hexavalent form as uranyl (UO_2_^2+^). The most common use of uranium is as fuel in nuclear power stations and for catalysis. Uranium finds its way into water through emissions from nuclear plants, as well as leeching from natural deposits, and the use of uranium containing fertilizers. In humans, uranium is linked to nephritis, which is inflammation of the kidneys. The World Health Organization set a provisional guideline value of 30 ppb, while Health Canada has a maximum allowable concentration of 20 ppb [140,141]. 

A colorimetric sensor for uranium(VI) based on a DNAzyme/AuNP system was developed by Lee et al. In this method, the DNAzyme-functionalized AuNPs aggregate to form a purple-colored solution. Once exposed to uranium(VI), the aggregate disassembles, and the solution turns red. This colorimetric shift can be correlated to the concentration of uranium(VI) in solution. Using this method, the ratiometric shift between 490 nm and 550 nm was correlated to the concentration of uranium(VI) in solution, and a linear dependence was observed. The sensor had a linear response from 50 nM to 2 µM (12 ppb to 476 ppb), with a detection limit of 12 ppb. When tested against other cations for interference, only uranium(VI) led to the de-aggregation of the functionalized AuNPs and, consequently, the colorimetric response [142].

A colorimetric method based on a peroxidase memetic assay for the detection of uranium(VI) was developed by Zhang et al. This method of detection is based on the inhibition of peroxidase-like activity that bovine serum albumin (BSA)-functionalized gold nanoclusters (AuNSs) naturally exhibit. When exposed to uranium(VI), the peroxidase-like activity is inhibited, and a colorimetric response is generated which can be correlated to the concentration of uranium(VI) in solution. Due to the peroxidase-like activity, an absorbance peak at 652 nm is present for the free functionalized AuNS. Once uranium(VI) is added to the solution, the peak decreases proportional to the concentration of uranium(VI) in solution. A linear correlation was seen for the decrease with a range of 12–160 µM (3 ppm to 38 ppm) with an *R*^2^ value of 0.9957. The limit of detection for this method was 1.86 µM (443 ppb). When compared to other cations that may interfere, uranium(VI) gave the largest response. Mercury(II) also gave a response, although it was not as significant as the uranium(VI). When tested in real water samples using the sample addition method, recovery for the method was 98% [143].

A fluorescent method for detecting uranium(VI) in water based on coprecipitation with calcium fluoride was developed by Perry et al. In this method, uranium was precipitated along with calcium fluoride; then, the precipitate was heated to 800 °C to calcify the sample. The samples were then pressed into pellets and analyzed using laser-induced fluorescence spectroscopy. The excitation wavelength was set to 488 nm for the test. The presence of uranium(VI) in the pellet led to a fluorescence enhancement at 530 nm. The enhancement was correlated to the concentration of uranium(VI) in solution, which yielded a linear range of 1 pM to 1 µM (0.2 ppt to 200 ppb) and a limit of detection of 0.04 pM (0.008 ppt), which is much lower than both WHO and Health Canada limits. Interference studies showed that the presence of other cations limits the linear range of the sensor, which only goes from 0.01 µM to 1 µM (2 ppb to 238 ppb) in a mixed solution. Analyses of real samples were in good agreement with their certified concentrations [144].

**Figure 18:**
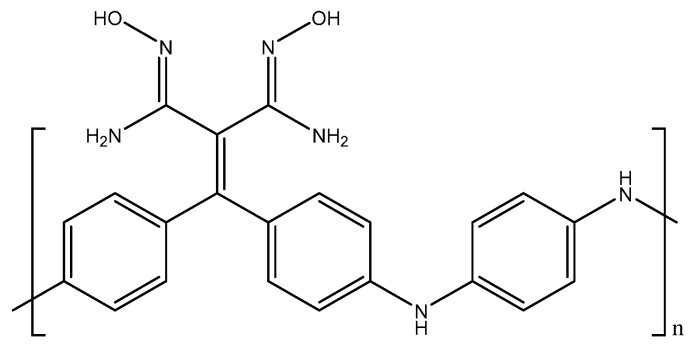
Structure of the amidoximated fluorescent polymer.

A fluorescent method based on an amidoximated polymer for the detection of uranium(VI) was developed by Ma et al. Upon binding to uranium(VI) in solution through the amidoxime groups on poly(diimino-2,2-dicyanoethylene), fluorescence of the polymer is quenched. This quenching is quantitative and can be correlated to the concentration of uranium(VI) in solution (Figure 18). 

When uranium(VI) was titrated into a solution of polymer in dimethylacetamide (DMA)/H_2_O (95/5, *v*/*v*), fluorescence quenching of the peak at 547 nm was seen proportional to the amount of uranium(VI) added to solution. A linear range was obtained going from 10 nM (the detection limit) to 150 nM (2 ppb to 36 ppb) with an *R*^2^ value of 0.986. When tested against other ions, no other ions gave the quenching response that the uranium(VI) did, making this a very selective method for uranium(VI) detection. When real water samples were tested using the sample addition method, recovery values were above 98%, and the values obtained were in good agreement with the standard ICP-MS method [145]. 

A selective PVC membrane for potentiometric detection of uranium(VI) was developed by Hassan et al. This membrane incorporates tris(2-ethylhexyl)phosphate (TEHP) as the electroactive material and sodium tetraphenylborate (NaTPB) as the ion discriminator selective toward uranium(VI). An Ag/AgCl electrode was used as the reference for this test. When the electrode is exposed to uranium(VI), the TEHP acts as the ionophore and binds to the uranium(VI), leading to a change in potential. Between the pH values of 2.8 and 3.6, a linear correlation between the potential and concentration was obtained, with a range of 20 µM to 10 mM (5 ppm to 2360 ppm). A limit of detection of 13 µM (3 ppm) was observed for this sensor. When comparing with other cations for interference, there was no response generated except for high concentrations of calcium(II), iron(II), and fluoride, which means that this sensor can be applicable for testing in soft water [146].

A CNT-modified glassy carbon electrode (GCE) for detection of uranium(VI) through ASV was developed by Golikand et al. When the deposition potential of −0.40 V is applied, the CNTs adsorb the reduced uranium from solution. Once the voltage is swept in the positive direction (using square wave), the uranium oxidizes at its oxidation potential, and goes back into solution. The current given by the stripping peak can be correlated to the concentration of uranium(VI) in solution. When testing the effect of pH on the stripping response, the optimal response was found to be at pH 4.4. When uranium(VI) was added to a solution of 0.2 M acetate buffer (pH 4.4) and 0.01 M magnesium(II), the oxidation peak had a peak current that increased with increasing concentrations of uranium(VI). A linear calibration was obtained with a range of 5 nM to 120 nM (1 ppb to 29 ppb) with an *R*^2^ value of 0.968. The detection limit for this method was determined to be 1 nM (0.2 ppb). When testing other cations and anions, their stripping peaks were quite distinct from the uranium(VI) peak, which makes this method very selective. When tested in real water samples, the sample addition method gave recoveries above 98% [147].

An SERS method for the detection of uranium(VI) was developed by Ruan et al. This method utilizes (aminomethyl)phosphonic acid (APA)-modified AuNPs as the active substrate. When uranium(VI) interacts with this substrate, an enhancement in the Raman peak can be seen, which can be correlated to the concentration of uranium(VI) in solution. Using the characteristic peak at 830 cm^−1^, the level of enhancement was correlated with the concentration of uranium(VI). The peak height increased linearly up to 2 ppm, where it began leveling off. This method gave a limit of detection of 200 ppb when tested. When using this method in simulated and in actual groundwater, uranium(VI) was still able to be detected at sub-ppm levels [148].

An SERS method based on functionalized silver colloids for the detection of uranium(VI) was developed by Trujillo et al. Glutathione is used to functionalize the silver colloid, which gives a Raman peak enhancement when interacting with uranium(VI) in solution. When the uranium(VI) interacts with the functionalized silver colloid, a new peak at 834 cm^−1^ arises, indicating that the uranium(VI) is bound to the colloid. The height of this peak is related to the concentration of uranium(VI) in the colloid solution, and a linear calibration can be obtained. The linear range of this method is 250–650 nM (60 ppb to 155 ppb), with an *R*^2^ value of 0.979. The detection limit for this method was determined to be 102 nM (24 ppb). When other cations were tested along with uranium(VI), a recovery of 88% was obtained, which, despite indicating a decrease in detection, is still suitable for use in a more complex matrix [149].

**Figure 19:**
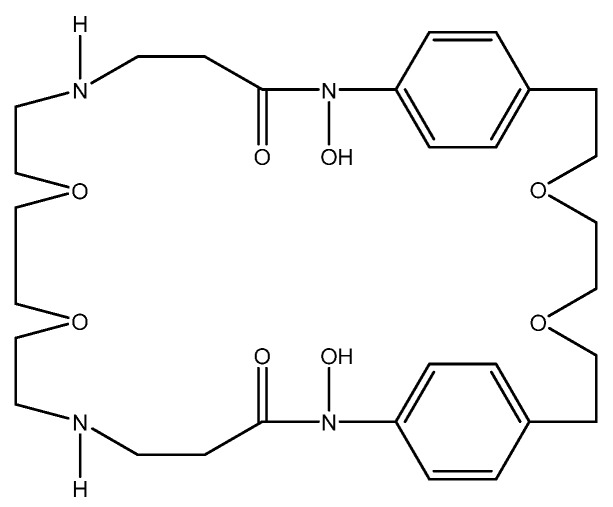
The structure of NHDTAHA.

An AAS method for the detection of uranium(VI) using a crown ether was developed by Agrawal et al. For this method, the uranium(VI) in solution is extracted through the use of 5,14-N,N′-hydroxyphenyl-4,15-dioxo-1,5,14,18-tetraaza hexacosane (NHDTAHA), and the extract is analyzed using GF AAS (Figure 19). 

The effect of pH was observed when extracting the uranium(VI). What was observed was that the maximum extraction for uranium(VI) occurred at pH 6–7. For the test, pH 6 was used. Many other ions were tested in the solution matrix to observe any effects on the recovery of uranium(VI), and none affected the extraction to a great degree. The limit of detection for this method was 10 ppb. When tested in real samples, the values obtained with this method were in good agreement with the certified values for the samples [150].

### 3.11. Zinc

Zinc is an essential element in human metabolism. Many enzymes in the body such as dehydrogenases, phosphatases, and more contain zinc [151]. The recommended daily intake of zinc is 0.3 mg/kg, with a maximum tolerance of 1 mg/kg of body weight. Adults require 15–22 mg of zinc per day [152]. When excess zinc is consumed, however, gastrointestinal issues (stomach cramps, vomiting, diarrhea) can occur within 3–12 h [153]. Zinc is also used for corrosion inhibition in various alloys, and is also used for galvanizing steel and iron, which is where the leaching of zinc into tap water is present [154]. As of present, there is no health guideline regarding zinc in drinking water; however, at 3 ppm, the water tends to be opalescent [155].

**Figure 20:**
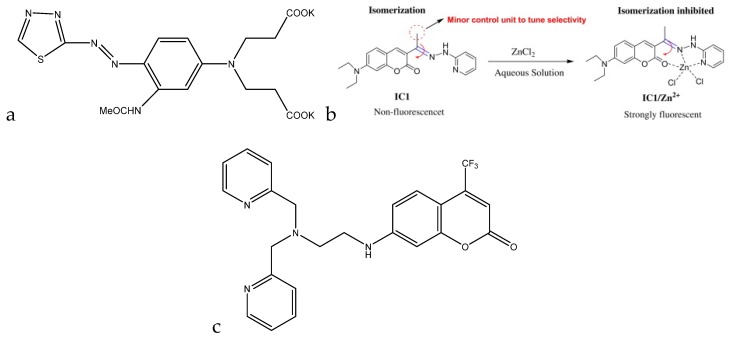
(**a**) Zinc(II)-selective hetarylazo derivative, (**b**) the detection mechanism of the sensor molecule (reprinted with permission from Reference [145]; Copyright 2012 Elsevier), and (**c**) structure of the fluorescent probe.

A colorimetric method of zinc(II) determination was developed by Kaur et al. using a hetarylazo derivative. The sensor that was developed is water-soluble and stable in pH >5, which makes it suitable for drinking water analysis (Figure 20a). 

When zinc(II) was titrated into the sensor-containing solution (pH 7.5 and 0.01 M HEPES in the presence of 0.15 M NaCl), the absorbance peak at 518 nm dropped in intensity, while the peak at 481 nm increased. This ratiometric change was used to create a calibration curve ranging from 0.006–0.015 M (392 ppm to 980 ppm). The sensor was also able to be reset using excess EDTA, which means that the sensor molecule is reusable again for another sample. Interference studies showed that cadmium, calcium, and magnesium did not affect sensor performance, which indicates selectivity to zinc(II) [156].

A fluorescence-based sensor utilizing an imine derivative was developed by Wu et al. The sensing mechanism is based on the C=N isomerization that occurs when the sensor is exposed to zinc(II) (Figure 20b). The sensor has an enhancement in fluorescence when exposed to zinc(II) in CH_3_CN/H_2_O buffer solution, as well as a shift in the emission peak from 519 nm to 509 nm after exposure to zinc(II) over a range of 1–10 μM (65 ppb to 654 ppb). The quantum yield of the fluorescence increased after exposure to zinc(II) from 0.014 to 0.26. When tested against other metal ions, only zinc(II) gave a significant enhancement to the fluorescence. Cadmium enhances the fluorescence as well (although not as much as the zinc), and all other metals either had no effect on the fluorescence or quenched the fluorescence [157].

A coumarin-derived fluorescent probe for zinc(II) based on photo-induced electron transfer was developed by Li et al. (Figure 20c). In 0.1 M phosphate buffer at pH 7 and at 30 °C, the fluorescence was quite weak at 510 nm for the sensor itself, with a quantum efficiency of 0.15. Once zinc(II) was titrated into the solution, the fluorescence intensity increased 13-fold, and the quantum efficiency went up to 0.47. The linear range for this sensor went from 0.44–10 μM (29 ppb to 654 ppb). Interferences were also tested to determine selectivity, and most did not give any fluorescence enhancement, even at high concentrations. Copper, cobalt, and nickel quenched the fluorescence rather than enhanced it, which is beneficial for the signal resolution of zinc. The only interfering ions for the sensor were mercury and cadmium, since they are in the same transition metal group. Their fluorescence enhancement, however, was not as significant, which indicates that the sensor is selective to zinc(II). The sensor was also tested at various pH levels, and it was found to perform the best between pH 6 and 9 (due to low pH protonating the sensor, and high pH forming Zn(OH)_2_) [158].

A thin film-based ASV method was developed by Ruecha et al. Both plastic film and paper were used as substrates, and the graphene/polyaniline (G/PANI) electrodes were deposited using drop casting and electrospraying. When testing with the two substrates, peak currents for the plastic film far exceeded that of the paper. Electrospraying the G/PANI electrode rather than drop casting also afforded higher sensitivity due to forming a more uniform film. The stripping mode used was a square wave ramp for all tests. At −1.31 V, the current showed a linear response with respect to the zinc(II) concentration, having a linear range of 1 ppb to 300 ppb. Interferences were tested for peak inhibition, and it was determined that there were no interferences from the range of −1.6 V to −0.5 V. The sensor was also tested in human serum and spiked to determine recovery. For each spike, the recovery remained within the range of 93.8% to 109.7%, which means that this system is reproducible [103].

A bismuth film electrode ASV method was developed by Kefala et al. For this method, three different substrates were considered: glassy carbon electrode (GCE), carbon paste, and impregnated graphite. In 0.1 M acetate buffer (4.5 pH), the GCE gave the highest stripping peaks, as well as the lowest noise; therefore, it was used for further analysis. When comparing bismuth film electrodes (BFEs) to the more common mercury film electrodes (MFEs), the sensitivity on the MFE was higher than the BFE. However, since the sensor was going to be used in situ, the BFE was used for analysis. At −1.31 V, the zinc stripping peak increased proportionally to the concentration of zinc(II), and a limit of detection of 0.7 ppb was obtained. The effects of surfactants (specifically Triton X-100) on the BFE were investigated, and it was found that the zinc(II) stripping peak decreased dramatically in its presence, which indicated that it would not be useful for urinalysis. Tap water was tested with this method and gave a result of 204 ppb, compared to 211 ppb obtained by AAS, which shows that this method is in good agreement with other analytical methods [159].

A method of zinc(II) determination in seawater using graphite furnace (GF) AAS was developed by Sturgeon et al. To ensure no background interference from the covolatilization of NaCl on the zinc(II) signal as the sample was being heated, NH_4_NO_3_ was added. This converted NaCl to NaNO_3_, which separated the zinc(II) peak from the background peak. Adding 10 mg per mL of NH_4_NO_3_ to a diluted 1:1 sample of seawater was found to be the optimal concentration. The graphite tube was also modified by making the walls at the ends of the tube thinner to improve detection limits. In the case of zinc(II), this allowed for a clean peak with minimal background or interfering peaks. Zinc(II) was analyzed through direct injection and a solvent preconcentration and extraction method. Using both methods, zinc(II) gave very similar results, only differing by 0.2 ppb. The LOD for this method was found to be 0.4 ppb [160].

A flow injection and preconcentration method for analyzing zinc(II) in seawater using FAAS was developed by Tony et al. The samples were adjusted to pH 3.0 ± 1.0 using NH_4_OAc. To ensure preconcentration, the zinc(II) was complexed with 5,7-dichlorooxine to adsorb onto the C_18_-bonded silica gel packed within the preconcentration column. The samples were run by themselves with a 2-ppb spike to ensure good recovery of adsorbed zinc(II). The working range was found to be 0 ppb to 50 ppb, with an LOD of 0.5 ppb. When spiked with 2 ppb of zinc(II), a recovery of 101% was obtained, and, when compared with samples with certified values, the analysis values came quite close, with the FAAS method finding 209 ppb in the MESS sample (certified 191 ± 17 ppb) and 165 ppb in the TORT sample (certified 171 ± 10 ppb) [161].

### 3.12. Simultaneous Detection of Analytes

Simultaneous detection of analytes is important for the wide-scale use of water quality assurance methods. Being able to detect more than one cation at the same time using only one method reduces the amount of instrumentation and workup required for the samples, and it allows one to collect data for multiple cations at the same time, which reduces workload.

**Figure 21:**
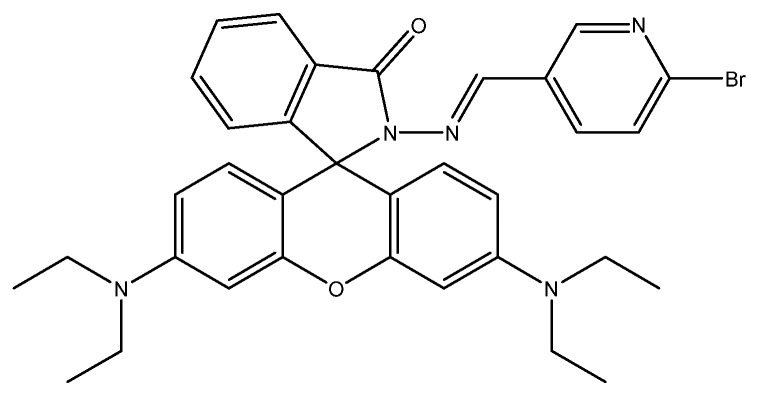
Structure of the rhodamine B-based chemosensor.

Rasheed et al. developed two colorimetric/fluorescent methods for the simultaneous detection of copper(II) and mercury(II) in aqueous media. The first method utilizes a rhodamine-based colorimetric probe for naked eye and colorimetric detection (Figure 21).

In acetonitrile/water (4:1 *v*/*v*, pH 7.2 with 10 mM HEPES) solution, a visual color change was seen going from colorless to pink for both cations. This occurs due to the spirolactam ring opening that occurs when copper(II) or mercury(II) bind at the azo group. This visual change was accompanied by an increase in absorbance at 559 nm for both cations. To distinguish the two cations, fluorescence spectroscopy was used. It was found that copper(II) did not induce fluorescence at 580 nm due to fluorescence quenching from the paramagnetic effect of copper(II)’s *d*^9^ system. Mercury(II), however, did induce fluorescence at 580 nm. The limits of detection for copper(II) and mercury(II) were 3.9 nM and 2.4 nM (0.2 ppb and 0.5 ppb), respectively. Both sensors had a linear range going from the limit of detection up to 10 μM (635 ppb for copper(II) and 2 ppm for mercury(II)). When tested against other cations for interference, no other cations interfered with the copper(II) or mercury(II) signal, making this quite a selective method [162]. The second method utilizes a chromogenic vesicle functionalized with a rhodamine derivative for the detection of copper(II) and mercury(II) in water. An alternating copolymer is functionalized with a rhodamine B derivative. When in contact with copper(II) or mercury(II), coordination occurs, causing a spirolactam ring opening of the xanthene backbone. This induces a color change from colorless to pink. When exposed to copper(II) and mercury(II) in aqueous solution, a color change from colorless to pink occurred, as well as an increase in the fluorescence at 558 nm and absorbance at 583 nm. The limits of detection for both copper(II) and mercury(II) were 30 nM and 20 nM (2 ppb and 4 ppb, respectively). The linear range of the sensors went from the detection limit up to 10 μM (635 ppb for copper(II) and 2 ppm for mercury(II)). In seawater samples, spiking the sample gave recovery values above 95%, making this a suitable sensor for more complex matrices [163].

A “periodic table style” paper device for simultaneous detection of copper(II), nickel(II), and chromium(VI) was developed by Li et al. This method utilizes chromatography paper which is coated in hydrophobic wax except for the detection area, which exposes the hydrophilic paper in the shape of the chemical symbol for the analyte. For the copper(II)-sensing portion, hydroxylamine (0.1 g/mL) in acetic buffer (6.3M, pH 4.3) was applied to the hydrophilic region. Then, 50 mg of bathocuproine in 1 mL of chloroform was added to the region along with 40 mg of polyethylene glycol (PEG) 400 (to preserve the hydrophilicity of the device). When exposed to copper(II) in solution, the “Cu” symbol printed onto the paper began changing color from yellow to brown. This color change occurred at 0.8 ppm, below which the color change was not discernible. For nickel(II), dimethylglyoxime dissolved in ethanol (80 mM) was applied as the sensor molecule. A solution of NaF followed by Na_2_S_2_O_3_ was then applied to prevent interference from copper(II). When exposed to nickel(II) in solution, the color of the “Ni” symbol changed from colorless to pink at 0.5 ppm. For chromium(VI), 1,5-diphenylcarbazide (1 mg/mL in 50% acetone) was applied as the sensor molecule, followed by H_2_SO_4_. When exposed to 0.5 ppm chromium(VI) in water, the color changed from colorless to purple, indicating the presence of chromium(VI). When interference tests were conducted with this device, no other ions seemed to interfere with the signal, and when pseudo-environmental samples were tested and compared to the standard ICP-AES method, the values obtained were in good agreement [164].

A method for simultaneous detection of mercury(II), lead(II), and copper(II) was developed by Guo et al. This method utilizes gold nanoparticles (AuNPs) functionalized with papain. The AuNPs, when in contact with either lead(II), mercury(II), or copper(II), display different colorimetric responses due to the differences in aggregation of the AuNPs. When aggregation was observed at various pH levels, a pH of 6 gave the biggest color change when exposed to mercury(II), with a red shift in the absorbance going from 524 nm to 626 nm. Below pH 6, aggregation was induced through the protonation of papain, whereas, at higher pH values, the hydroxyl groups interacted with the cations, preventing aggregation. When tested with mercury(II), a visual color change from pink to blue was observed, accompanied by the previously mentioned red shift in the absorbance spectrum. The lowest concentration to induce a color change was 4 μM (802 ppb), which is much higher than the WHO maximum limit. Copper(II) and lead(II) also induce aggregation of the AuNPs, albeit at lower intensity. When tested against other cations, the AuNP solution remained pink, indicating that aggregation was not induced. When testing a real water sample, spiking the water gave good recovery and induced a color change, making this method feasible for more complex matrices [165].

An ASV method for simultaneous detection of lead(II) and cadmium(II) was developed by Promphet et al. This method uses graphene/polyaniline/polystyrene (G/PANI/PS) screen-printed carbon electrodes for electrochemical detection. To modify the surface of the electrode, G/PAN/PS nanoporous fibers were electrospun onto the electrode. This was used as the working electrode, along with a Pt wire and the counter electrode, and a reference Ag/AgCl electrode. The cadmium(II) and the lead(II) ions were deposited onto the electrode by applying a −1.2 V potential, and bismuth(III) ions were used to improve the response of the electrode. At a bismuth(III) concentration of 900 ppb, cadmium(II) and lead(II) voltammograms were able to be distinguished. Both cadmium(II) and lead(II) had a linear range going from 10 ppb to 500 ppb, with *R*^2^ values of 0.992 and 0.991, respectively. The limits of detection were 3.3 ppb for lead(II) and 4.43 ppb for cadmium(II). When tested against other ions for interference, the voltammograms did not interfere with the cadmium(II) or lead(II) signal, with the exception of copper(II) and zinc(II) at very high concentrations. When measuring real samples and comparing to the standard ICP-AES method, the values obtained were in good agreement with each other [166].

An electrochemical method for the simultaneous determination of silver(I) and mercury(II) was developed by Miao et al. This method uses DNA1-modified Fe_3_O_4_@Au nanoparticles. Upon exposure to either silver(I) or mercury(II), the DNA1 immobilized on the nanoparticle hybridizes either to DNA2 (labeled with ferrocene) or DNA3 (labeled with methylene blue) based on whether it binds to silver(I) or mercury(II), respectively. This occurs through the cytosine–Ag^+^–cytosine bond and the thymine–Hg^2+^–thymine bond between the DNA strands. Square wave voltammetry is then used to quantify the amount of silver(I) or mercury(II) in solution, with a magnetic glassy carbon electrode as the working electrode, a Pt wire as the counter electrode, and an SCE reference. The presence of aqueous silver(I) and mercury(II) resulted in a specific current peak for silver at 0.36 V and for mercury(II) at −0.39 V vs. SCE. The linear range for silver(I) went from 10 to 150 nM (1 ppb to 16 ppb), and that for mercury(II) went from 10 nM to 100 nM (2 ppb to 20 ppb), with *R*^2^ values of 0.982 and 0.985, respectively. The limit of detection for silver(I) was 0.37 ppb, and that for mercury(II) was 0.34 ppb. When testing against other ions for interference, none interfered with the silver(I) or the mercury(II) signal. In real water samples, spiking the water samples showed good recovery, and the values obtained were in good agreement with the standard AAS method [167]. 

## 4. Conclusions

In this review, the importance of the detection of cations in drinking water was discussed with respect to the effects that various cations have on human physiology, as well as the sources of these cations in water. A number of methods for analysis were investigated in terms of all the analytes presented, as well as modifications to those methods specific to each analyte. Examples of the methods discussed were presented for each analyte, along with their applicability to real life water quality detection. While some methods were more simplistic in their approach (colorimetric yes/no assay), others were much more complex (spectroscopy), which was reflected in the accuracy of the results obtained. Overall, it can be concluded that lighter transition metals (copper, zinc, chromium, nickel) are detectable using relatively simple methods such as colorimetry or fluorescence with sufficiently low detection limits (below regulation). This could be due to the fact that lighter metals are more reactive and interact more readily with colorimetric and fluorescent indicators; this could also be due to the higher limits compared to the heavier metals. Heavier metals (lead, mercury, cadmium, uranium) tend to have higher detection limits when using colorimetric methods due to their lower reactivity, which means that more complex methods such as AAS and ASV are more appropriate to measure these elements. In general, mechanical and optical methods have higher detection limits with large linear ranges, making them appropriate for high-range detection of analytes. Methods such as potentiometry, ASV, SERS, and AAS are better at detecting low-range analytes due to the high sensitivity and lower linear range compared to optical and mechanical methods. For the future, one must look toward sensor networks for continuous in situ monitoring of water supplies and the detection of short-term changes, automation of sample preparation prior to analysis, and analysis of elemental species (ionic salt vs. organic, complexes, changes in oxidation state) [168].

The field of water quality monitoring is very diverse, and, with many other analytes and parameters to consider, the opportunities for development in this area are limitless.

## Figures and Tables

**Table 1:** Guidelines for water quality and standard detection methods.

	Canada ^1^	US ^2^	Europe ^3^	WHO ^4^	Standard Method *
pH	7.0–10.5	6.5–8.5	6.5–9.5	-	
Barium	1 ppm	2 ppm	-	1.3 ppm	ICP-MS ^a^ (200.8, 7440-39-3) ICP-AES ^b^ (200.7, 7440-39-3) FAAS ^c^ (7000B, 7440-39-3)
Cadmium	5 ppb	5 ppb	5 ppb	3 ppb	ICP-MS (200.8, 7440-43-9) ICP-AES (200.7, 7440-43-9) FAAS (7000B, 7440-43-9)
Chromium	50 ppb	100 ppb	50 ppb	50 ppb	ICP-MS (200.8, 7440-47-3) ICP-AES (200.7, 7440-47-3) FAAS (7000B, 7440-47-3)
Copper	2 ppm	1.3 ppm	2 ppm	2 ppm	ICP-MS (200.8, 7440-50-8) ICP-AES (200.7, 7440-50-8) FAAS (7000B, 7440-50-8)
Hardness	-	-	-	-	ICP-AES (200.7, 7440-70-2 (Ca), 7439-95-4 (Mg)) FAAS (7000B, 7440-70-2 (Ca), 7439-95-4 (Mg))
Lead	5 ppb ALARA	15 ppb ALARA	10 ppb	10 ppb	ICP-MS (200.8, 7439-92-1) ICP-AES (200.7, 7439-92-1) FAAS (7000B, 7439-92-1)
Mercury	1 ppb	2 ppb	1 ppb	6 ppb	ICP-MS (200.8, 7439-97-6) ICP-AES (200.7, 7439-97-6)
Nickel	-	-	20 ppb	70 ppb	ICP-MS (200.8, 7440-02-0) ICP-AES (200.7, 7440-02-0) FAAS (7000B, 7440-02-0)
Silver	-	-	-	100 ppb REC	ICP-MS (200.8, 7440-22-4) ICP-AES (200.7, 7440-22-4) FAAS (7000B, 7440-22-4)
Uranium	20 ppb	30 ppb	-	30 ppb	ICP-MS (200.8, 7440-61-1)
Zinc	5 ppm	-	-	-	ICP-MS (200.8, 7440-66-6) ICP-AES (200.7, 7440-66-6) FAAS (7000B, 7440-66-6)

^1^ Health Canada: Guidelines for Canadian Drinking Water Quality. 2018. ^2^ United States Environmental Protection Agency (EPA): National Primary Drinking Water Regulations. 2018. ^3^ European Union: European Drinking Water Directive. 2019. ^4^ World Health Organization: Guidelines for Drinking Water Quality. 2017. * Listed are the EPA methods in this format: [method] ([method number], [CASRN]). ^a^ Inductively coupled plasma mass spectrometry. ^b^ Inductively coupled plasma atomic emission spectroscopy. ^c^ Flame atomic absorption spectroscopy. ALARA = as low as reasonably achievable; REC = recommended
